# Chondrogenic Differentiation of Defined Equine Mesenchymal Stem Cells Derived from Umbilical Cord Blood for Use in Cartilage Repair Therapy

**DOI:** 10.3390/ijms19020537

**Published:** 2018-02-10

**Authors:** Mélanie Desancé, Romain Contentin, Lélia Bertoni, Tangni Gomez-Leduc, Thomas Branly, Sandrine Jacquet, Jean-Marc Betsch, Agnès Batho, Florence Legendre, Fabrice Audigié, Philippe Galéra, Magali Demoor

**Affiliations:** 1 Normandie University, UNICAEN, BIOTARGEN, 14000 Caen, France; melanie359@hotmail.fr (M.D.); romaincontentin@hotmail.fr (R.C.); tangnigl@free.fr (T.G.-L.); tbranly@gmail.com (T.B.); Agnes.BATHO@efs.sante.fr (A.B.); florence.legendre@unicaen.fr (F.L.); 2 Center of Imaging and Research on Locomotor Affections in Equines, Ecole Vétérinaire d’Alfort, Université Paris-Est, 14430 Goustranville, France; lelia.bertoni@vet-alfort.fr (L.B.); sandrine.jacquet@vet-alfort.fr (S.J.); fabrice.audigie@vet-alfort.fr (F.A.); 3 Clinique Vétérinaire Equine de Méheudin, Méheudin, 61150 Ecouché, France; jmbetsch@cvem.fr; 4 EFS Caen, 14000 Caen, France

**Keywords:** mesenchymal stem cells, umbilical cord blood, horse, cartilage engineering, chondrogenesis, extracellular matrix, RNA interference

## Abstract

Cartilage engineering is a new strategy for the treatment of cartilage damage due to osteoarthritis or trauma in humans. Racehorses are exposed to the same type of cartilage damage and the anatomical, cellular, and biochemical properties of their cartilage are comparable to those of human cartilage, making the horse an excellent model for the development of cartilage engineering. Human mesenchymal stem cells (MSCs) differentiated into chondrocytes with chondrogenic factors in a biomaterial appears to be a promising therapeutic approach for direct implantation and cartilage repair. Here, we characterized equine umbilical cord blood-derived MSCs (eUCB-MSCs) and evaluated their potential for chondrocyte differentiation for use in cartilage repair therapy. Our results show that isolated eUCB-MSCs had high proliferative capacity and differentiated easily into osteoblasts and chondrocytes, but not into adipocytes. A three-dimensional (3D) culture approach with the chondrogenic factors BMP-2 and TGF-β1 potentiated chondrogenic differentiation with a significant increase in cartilage-specific markers at the mRNA level (*Col2a1*, *Acan*, *Snorc*) and the protein level (type II and IIB collagen) without an increase in hypertrophic chondrocyte markers (*Col10a1* and *Mmp13*) in normoxia and in hypoxia. However, these chondrogenic factors caused an increase in type I collagen, which can be reduced using small interfering RNA targeting *Col1a2*. This study provides robust data on MSCs characterization and demonstrates that eUCB-MSCs have a great potential for cartilage tissue engineering.

## 1. Introduction

Osteoarthritis is the most common musculoskeletal disease in horses and is characterized by varying degrees of lameness, the presence of joint effusion, the occurrence of osteophytes, an increase in bone density, and the reduction of joint space in more advanced cases [1]. In a mobile joint, articular cartilage is a highly specialized connective tissue, avascular and non-innervated [2]. This articular cartilage covers bone ends in joints to prevent friction and protect the bone from damage caused by abnormal mechanical loading. Articular cartilage is composed of chondrocytes that synthesize an intricate extracellular matrix (ECM). The ECM consists of water linked to proteoglycans, such as aggrecans and type II collagen networks [3]. Due to the poor self-repair capacity of cartilage, cartilage engineering is a new therapeutic strategy to treat osteoarthritic or trauma cartilage defects in humans. In racehorses, locomotor disorders are the leading cause of reduced performance and the early termination of racehorse careers [4]. The anatomical, cellular, and biochemical properties of cartilage are similar between humans and horses [5], and cartilaginous defects associated with intense physical activity have recently been recognized as similar in both species. Therefore, the horse is an excellent model for the development of cartilage engineering [6].

Autologous chondrocyte implantation (ACI) was first described in 1994 [7] and was evaluated in the equine model in 2011 [8]. This technique involves harvesting cartilage tissue from the patient, isolating chondrocytes from the donor tissue, and expanding the cells in vitro to implant the cells directly in the cartilage lesion. However, ACI treatment leads to undesirable fibrocartilage formation mainly due to the dedifferentiation of chondrocytes during expansion [9]. A new approach involves the use of mesenchymal stem cells (MSCs), which have a high potential for chondrocyte differentiation and thus cell-based treatment of cartilage defects.

MSCs were first identified in bone marrow [10] and have since been isolated from a variety of other tissues in humans and horses, such as umbilical cord blood (UCB), umbilical cord matrix (Wharton’s jelly), adipose tissue, dental pulp, peripheral blood, and synovial fluid [11,12,13,14]. The International Society for Cellular Therapy (ISCT) stipulates the minimal criteria for defining human MSCs: adherence to a plastic surface, expression of the CD105, CD73, and CD90, but not CD45, CD34, CD14, CD19, and HLA-DR surface molecules, and the ability to differentiate into osteoblasts, adipocytes, or chondrocytes in vitro [15]. However, in other species such as the horse, the presence of surface markers can differ from those in human MSCs. In equine umbilical cord blood-derived MSCs (eUCB-MSCs), CD29, CD44, CD90, and CD105 are expressed [16], but not CD73 [17]. However, CD105 expression is varying in eUCB-MSCs.

In horses and humans, MSCs derived from bone marrow are the most studied and are often considered the gold standard [10]. However, the sampling procedure is invasive and it has been shown that MSCs derived from human bone marrow taken from older patients have a limited lifespan and show an accelerated cell senescence [18]. Comparative analyses of proliferation and multilineage capacity have shown that human MSCs derived from UCB have greater potential than MSCs derived from bone marrow or adipose tissue [19,20]. Moreover, supplementation with fibroblast growth factor-2 (FGF-2) during MSCs expansion maintains the differentiation potential and increases proliferative capacity [21,22]. Owing to their abundance and the painless non-invasive harvesting procedure, UCB-derived MSCs (UCB-MSCs) are a promising alternative source to bone marrow.

Many studies have attempted to differentiate MSCs into a chondrogenic lineage in vitro. This chondrocyte differentiation is based, in part, on the use of chondrogenic factors. The growth factors of the transforming growth factor-β (TGF-β) superfamily induce chondrogenesis in MSCs in vitro [23,24]. The three TGF-β (TGF-β1, -β2, and -β3) can induce chondrogenesis [25]. In equine MSCs derived from bone marrow and adipose tissue, chondrogenesis is predominantly induced with TGF-β1. However, only cells derived from bone marrow appear to synthesize type II collagen [26].

Chondrogenesis is enhanced when the bone morphogenetic proteins (BMPs) are added along with TGF-βs [27,28]. In human MSCs, chondrogenesis induced by TGF-β1 and BMP-2 leads to high expression of chondrogenic genes (*COL2A1*, *SOX9*, *ACAN*) [29]. The effect of BMP-2 has not yet been assessed in equine MSCs chondrogenesis experiments. 

The use of biomaterials is a key component in the success of cartilage engineering, because their chemical and physical properties influence the quality of the neo-synthesized tissue. Among the various biomaterials available, type I/III collagen sponges show efficient chondrogenic differentiation of human UCB-MSCs when used with BMP-2 and TGF-β1, leading to a strong increase in mature chondrocyte-specific markers (*COL2A1*, *COL2A*, *ACAN*) and cellular phenotype stability in the neo-tissue [30]. 

In addition to the specific markers of cartilage, TGF-β also induces the in vitro expression of type X collagen, a hypertrophic chondrocyte and osteoarthritis marker [31]. The challenge in the use of MSCs as a source of cells for cartilage engineering is to prevent hypertrophic outcomes [32,33]. Hypoxia plays a key role in this process and promotes the stabilization of a chondrocyte phenotype, whereas cells cultured in normoxia produce a hypertrophic phenotype [28].

Today, research is mainly based on the use of undifferentiated MSCs for the treatment of the equine locomotor disorders related to inflammation. Clinical application of eUCB-MSCs has shown that cell transplantation can reduce the core lesion in cases of inflammation of the superficial digital flexor tendon [34]. For cartilage damage, research with MSCs in an equine model has consisted in the study of the effects of intra-articular injection of MSCs [35]. Allogeneic and autologous injections of equine bone marrow-derived-MSCs in fetlock joints do not cause immune responses [36]. Injections of eUCB-MSCs, following lipopolysaccharide injection, reduce joint inflammation [37]. Evaluation of cartilage repair with injection of equine MSCs in polymerizing autogenous fibrin vehicle was performed in horses with 15-mm cartilage lesions in femoropatellar joints [38]. After 30 days, biopsy shows that MSC-implanted defects contain an increase of fibrous tissue predominantly composed of type II collagen. However, after eight months, no significant differences were observed between stem cell-treated joints and non-treated joints.

The promising results obtained with equine MSCs for tendon and joint disease in the equine model offer a glimpse of a possible transposition of these therapeutic strategies to human preclinical studies [39]. However, knowledge is lacking regarding the level of analogy between equine MSCs and their human counterparts. Therefore, a more thorough phenotypic characterization of equine MSCs is required to assess their therapeutic potential and guide the development of new therapeutic strategies for locomotor disorders in horses.

In this study, we characterized equine UCB-MSCs and determined their potential for cartilage tissue engineering using type I/III collagen sponges as the three-dimensional (3D) scaffold and chondrogenic factors under two oxygen conditions (normoxia and hypoxia). MSCs characterization was performed by evaluating proliferation, multilineage capacity, and immunophenotype and by investigating the expression of different genes involved in cellular senescence, stemness, and proliferation. The quality of the newly synthesized matrix in vitro was assessed by studying cartilage-specific and non-specific markers as well as markers of hypertrophy. Finally, to stabilize the phenotype of differentiated UCB-MSCs, the use of small interfering RNA (siRNA) targeting *Col1a2* mRNA was tested.

## 2. Results

### 2.1. Equine Umbilical Cord Blood-Derived Mesenchymal Stem Cells (eUCB-MSCs) Isolation

**Figure 1:**
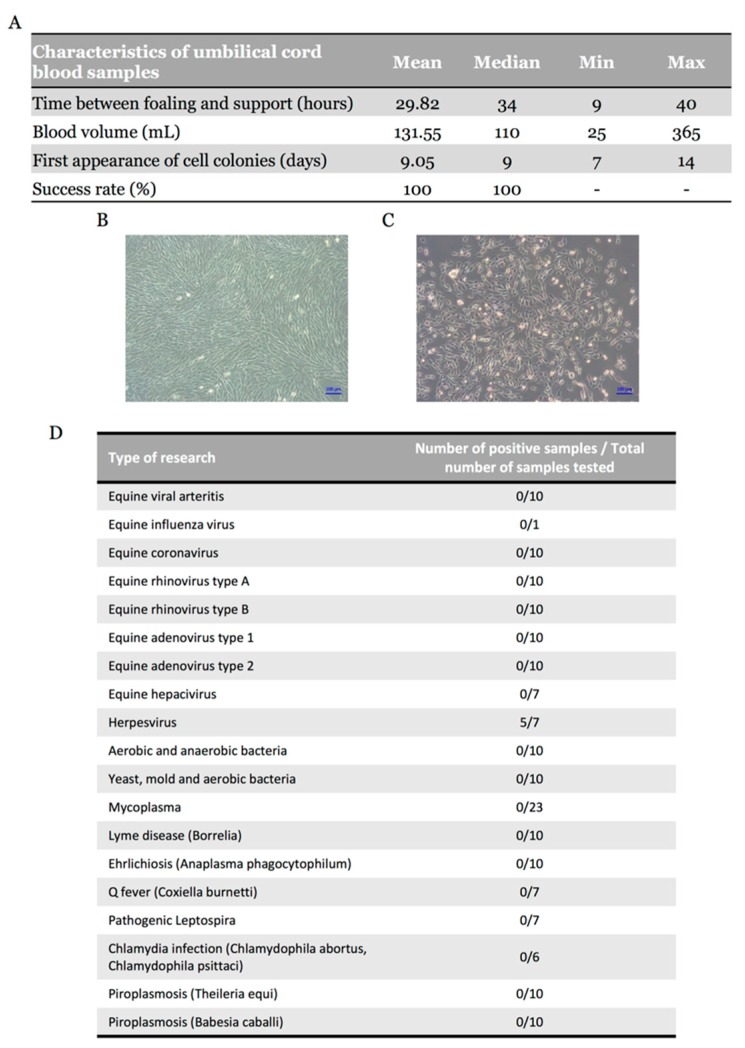
Morphology of mesenchymal stem cells (MSCs) and characteristics of equine umbilical cord blood (eUCB) samples. (**A**) Characteristics of equine umbilical cord blood samples (*n* = 22); (**B**) Phase-contrast microscopy of adherent UCB-MSCs in culture (magnification ×10) at passage two and at passage zero (**C**); (**D**) Validation of safety of equine UCB-MSCs. The different analyses were performed on a sample of trypsinized cells resuspended in the culture medium which was in contact with the cells for at least two days.

UCB samples were collected from 24 mares with normal parturition. Equine MSCs were successfully isolated in 22 UCB samples; the two other UCB samples had been processed more than 40 h after collection, hindering the emergence of colonies. The time between foaling and sample processing was on average 29.82 h with a volume of blood ranging from 25 to 365 mL (median, 110 mL) (Figure 1A). Spindle-shaped fibroblast-like adherent cells were observed in all 22 samples (Figure 1B), representing an isolation success rate of 100% with the first appearance of cell colonies after nine days of culture (Figure 1C). These results suggest that isolation success does not depend on UCB volume, but on processing time, which must not exceed 40 h. Cryopreservation was performed at each passage, until the fifth passage (P5) (Supplementary Table S1). To ensure the safety of isolated cells, bacteriological and virological analyses were also carried out (nine viral genera, eight bacterial genera, and two protozoa were targeted) and showed positive samples only for Herpesvirus (71%) (Figure 1D).

### 2.2. Growth Profiling and Cellular Senescence

**Figure 2:**
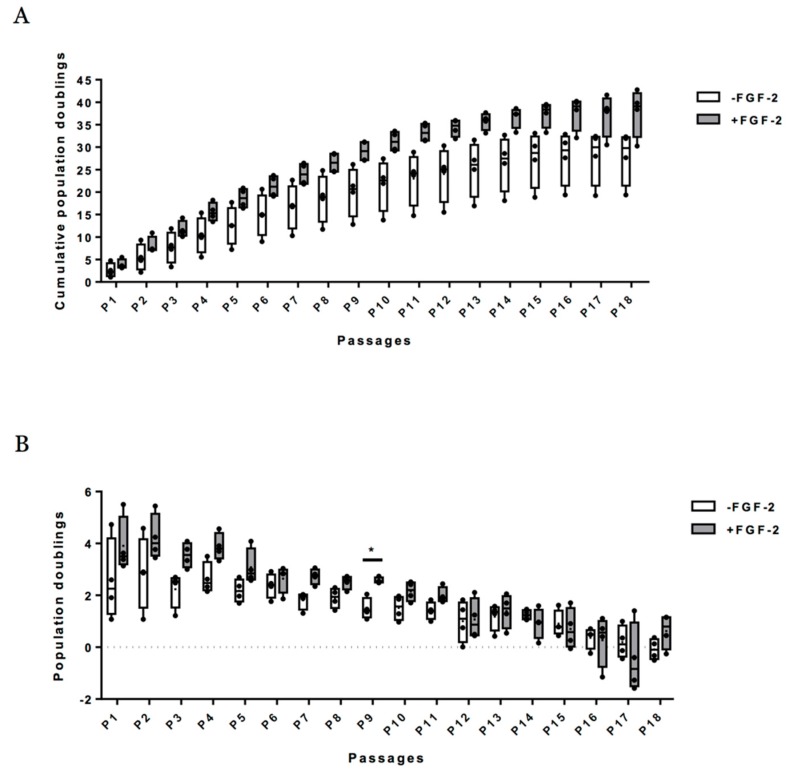
Effect of fibroblast growth factor-2 (FGF-2) on proliferation capacity of equine umbilical cord blood-derived mesenchymal stem cells (eUCB-MSCs). (**A**) Mean value of cumulative population doublings and (**B**) mean value of population doublings. Population doublings were determined at each passage of the adherent eUCB-MSCs cultured with (+FGF-2) or without FGF-2 (−FGF-2). Graph represents the mean ± standard deviation (*n* = 4). Statistically significant differences among eUCB-MSCs between the two culture media at each passage were determined using multiple *t*-test (* *p* < 0.05).

**Figure 3:**
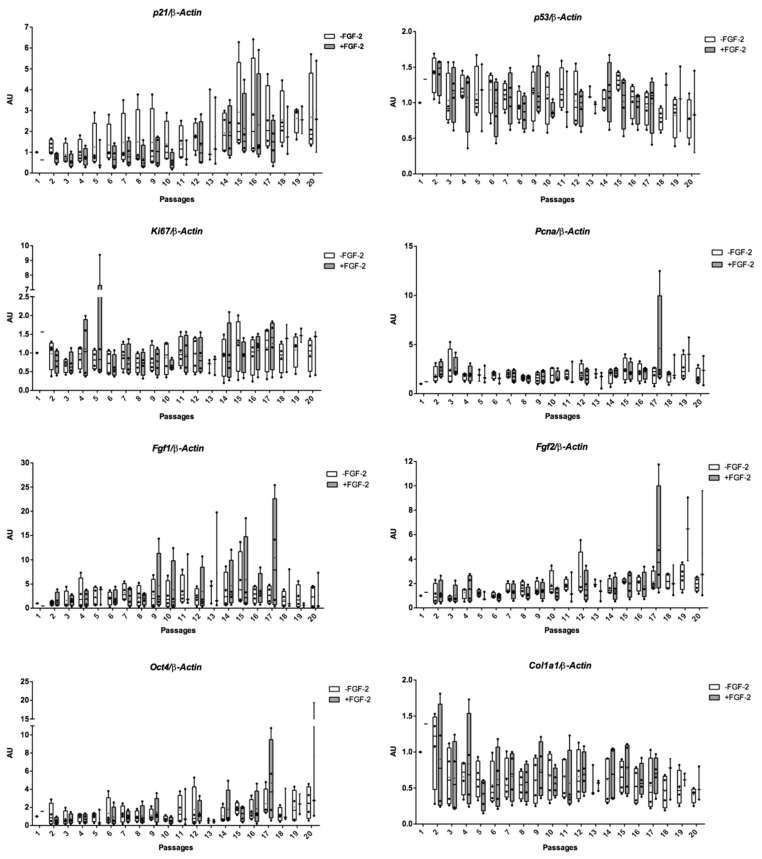
Expression of senescence (*p21*), cell cycle (*p53*), proliferation (*Pcna*, *Ki67*, *Fgf1*, *Fgf2*), and stemness markers (*Oct4*, *Col1a1*) in equine umbilical cord blood-derived mesenchymal stem cells (eUCB-MSCs) following long-term culture with fibroblast growth factor-2 (FGF-2). eUCB-MSCs were cultured in monolayer in the absence (−FGF-2) or presence of 5 ng/mL of FGF-2 (+FGF-2). All results were normalized to *β-Actin* mRNA expression, compared with eUCB-MSCs cultured in monolayer at the first passage in the absence of FGF-2. The results are presented as the relative expression of each gene. Box plot represent four independent experiments performed in triplicate. Statistically significant differences between the two culture media at each passage were determined using multiple *t*-test. Statistically significant differences between each passage were determined using the Kruskal-Wallis test. AU: arbitrary units.

Figure 2 summarizes calculated cumulative population doubling (PD) versus passage number without the FGF-2 growth factor and with FGF-2 (up to 18 passages). An increase in cumulative PD was observed in both expansion conditions with an average of 37.79 ± 5.36 at P18 with FGF-2 and an average of 27.82 ± 6.02 at P18 without FGF-2 (Figure 2A). The PD was higher in the presence of FGF-2 than in the absence of FGF-2 (Figure 2B). However, differences between conditions were not significant for a given passage, except at P9. With and without FGF-2, cumulative PD is slowed down after P15 (Figure 2A,B and Figure S1). Cellular senescence was assessed by analyzing *p21* and *p53* mRNA levels at each passage on eUCB-MSCs expanded with or without FGF-2. There were no significant differences between the two expansion conditions, although *p21* appeared to increase in both conditions after P15 (Figure 3). This result can be attributed to the slowing cumulative PD described above. Regarding proliferation markers (*Ki67*, *Pcna*, *Fgf1*, and *Fgf2*), the stemness marker (*Oct4*), or the MSCs lineage marker (*Col1a1*), no significant differences were observed between the two culture conditions or between passages (Figure 3).

### 2.3. MSCs Multipotential Capacity

**Figure 4:**
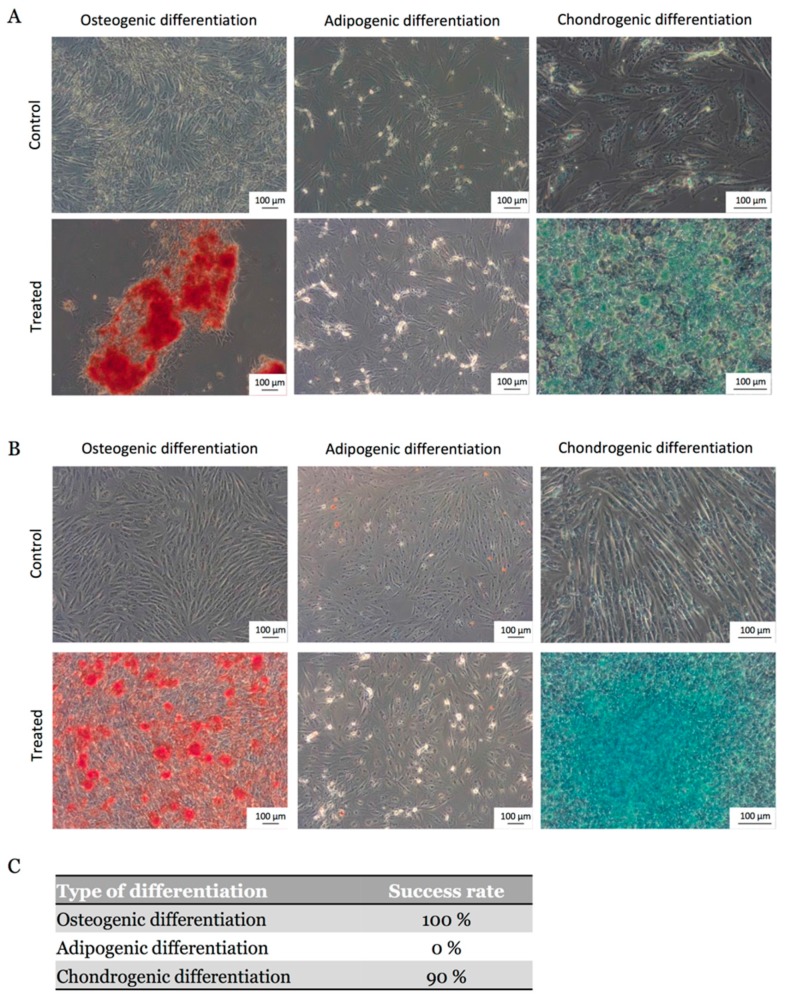
Osteogenic, adipogenic, and chondrogenic differentiations of equine umbilical cord blood-derived mesenchymal stem cells (eUCB-MSCs). (**A**) Differentiations were performed at passage four after amplification without FGF-2; (**B**) Differentiations were performed at passage four after amplification with FGF-2; (**C**) Success rate for the differentiation of eUCB-MSCs into osteoblasts, adipocytes, and chondrocytes (*n* = 10). After culture in osteoblastic induction medium, calcium mineralization was demonstrated by Alizarin Red S staining (magnification ×10). After adipogenic induction and incubation in the maintenance medium, no lipid droplets in the cytoplasm were observed with Oil Red O staining (magnification ×10). After chondrogenic induction, sulfated proteoglycans of the neo-synthetisized matrix were demonstrated by Alcian blue staining (magnification ×20).

To investigate the multilineage potential of eUCB-MSCs, osteogenic, adipogenic, and chondrogenic induction assays were performed in vitro with and without FGF-2 during the expansion phase on 10 eUCB-MSC samples. All eUCB-MSCs included in the study successfully differentiated after P4 into an osteogenic lineage and 90% of them into a chondrogenic lineage, regardless of the presence or absence of FGF-2. After 21 days in osteogenic induction medium, Alizarin Red S staining revealed mineralization via calcium deposition (Figure 4A,B). Chondrogenesis was induced in monolayer and Alcian blue staining indicated the presence of acidic polysaccharides such as glycosaminoglycans. The staining was stronger for eUCB-MSCs expanded in the presence of FGF-2 (Figure 4B). Regarding adipogenic differentiation, no lipid droplets were detected in the cytoplasm by Oil Red O staining. Thus, eUCB-MSCs have a partial mesenchymal lineage differentiation ability (Figure 4C).

### 2.4. eUCB-MSCs Surface Phenotype Expression

**Figure 5:**
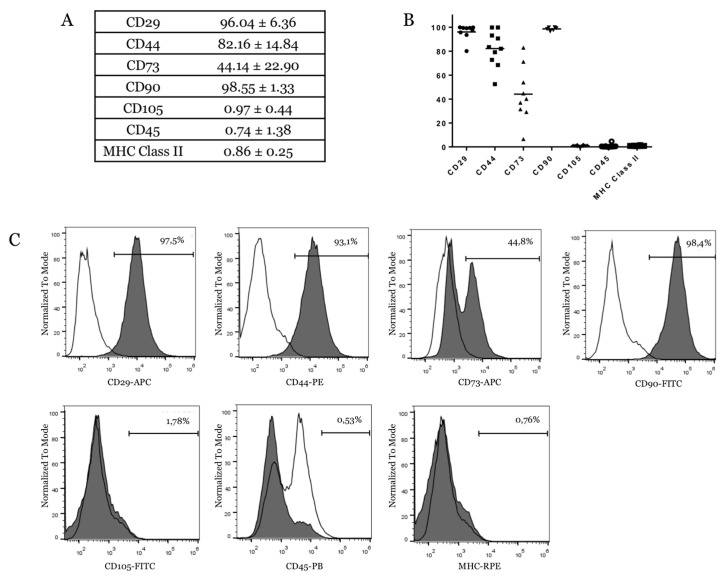
Immunophenotype of equine umbilical cord blood-derived mesenchymal stem cells (eUCB-MSCs). (**A**,**B**) Expression of surface markers of eUCB-MSCs at passage four. The table and the graph show the mean values of the percentage of positive cells ± standard deviation for the total number of cells analyzed (*n* = 10 for CD44, CD105, CD45, and major histocompatibility complex (MHC) Class II. *n* = 9 for CD29 and CD73. *n* = 6 for CD90). In panel **B**, each sample for the different CDs studied is represented by a symbol (black dot or arrowhead or square; (**C**) Immunophenotype of eUCB-MSCs at passage four. Each histogram is a representative result of at least six eUCB-MSCs samples. Lines with empty surface show the IgG (or IgM for CD90) isotype control response and lines with grey surface are for the samples.

The characterization of cell-surface markers on isolated eUCB-MSCs was performed using flow cytometry at P4. The results showed that cells were negative for the expression of the hematopoietic marker CD45 and lacked major histocompatibility complex (MHC) Class II. eUCB-MSCs were positive for several MSCs markers including CD29, CD44, and CD90 (Figure 5A). CD73 expression was donor-dependent with an expression ranging from 6.52 to 71.2% (Figure 5B), and it was expressed by only a part of the cell population (Figure 5C). eUCB-MSCs lacked CD105, a surface marker related to a major glycoprotein of the vascular endothelium and component of the TGF-β receptor complex.

### 2.5. Expression Analysis of Chondrogenic Markers and Cartilage Non-Specific Genes in eUCB-MSCs in 3D Scaffolds

Chondrogenic differentiation was induced in eUCB-MSCs seeded in collagen sponge scaffolds and cultured in the presence or absence of BMP-2 and TGF-β1 for seven, 14, 21, and 28 days in normoxia or in hypoxia. The evaluation of the chondrogenic differentiation was assessed by RT-qPCR for genes that encode proteins characteristic of native hyaline cartilage: *Col2a1*, *Acan*, and *Snorc*. *Col1a1* and *HtrA1* encode respectively a non-specific protein and a protease induced in osteoarthritis (OA) cartilage. *Col10a1* and *Runx2* encode markers of hypertrophic cartilage, and *Osteocalcin* (*Ostc*) of osteoblastic maturation. *Mmp1*, *Mmp3*, and *Mmp13* encode enzymes of cartilage degradation.

**Figure 6:**
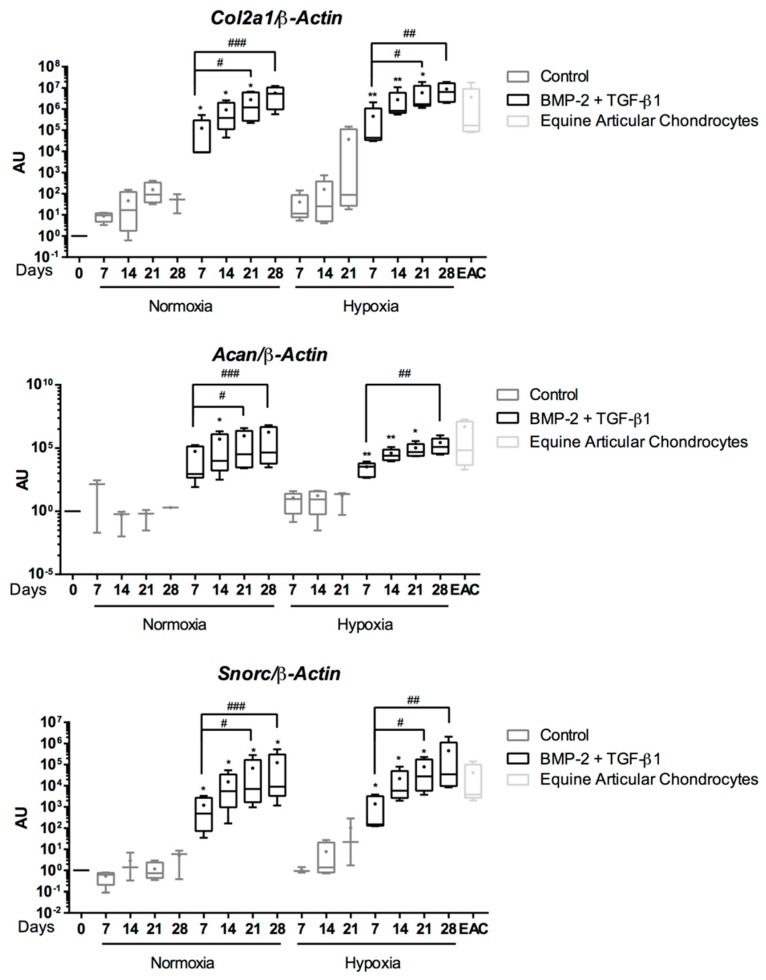
Effect of chondrogenic factors and oxygen tension on the gene expression of cartilage-specific markers during the chondrogenic differentiation of equine umbilical cord blood-derived mesenchymal stem cells (eUCB-MSCs). eUCB-MSCs were cultured in type I/III collagen sponges for seven, 14, 21, and 28 days in normoxia or in hypoxia, in the absence (control), or in the presence of 50 ng/mL of BMP-2 and 10 ng/mL of TGF-β1 (BMP-2 + TGF-β1). Real-time RT-PCR analysis of relative mRNA expression of the indicated genes (*Col2a1*, *Acan*, and *Snorc*) is shown. Gene expression was normalized to the *β-Actin* mRNA expression, compared with eUCB-MSCs cultured in monolayer at the fourth passage (Day 0). Equine articular chondrocyte (EAC) mRNAs were used as a positive control. Box plots represent five independent experiments performed in triplicate. Statistically significant differences between time points with the same culture medium were determined using the Friedman test (^#^ *p* < 0.05, ^##^ *p* < 0.01, ^###^ *p* < 0.001). Statistically significant differences between the culture medium at the same time point were determined using the two-tailed Mann–Whitney test (* *p* < 0.05, ** *p* < 0.01). Comparison of the differences between normoxia and hypoxia was performed using the two-tailed Mann–Whitney test, and no statistical significant differences were observed. AU: arbitrary units.

In the absence of growth factors, at the mRNA level, eUCB-MSCs seeded in a 3D scaffold exhibited a slight increase in *Col2a1*, *Acan*, and *Snorc* mRNA levels during culture in hypoxia but not in normoxia. However, no statistical differences were observed compared with undifferentiated cells (Control) (Figure 6). It should also be noted that in the absence of growth factors, some cell cultures did not go to their term, which explains the absence of data, particularly at D28 in hypoxia. When BMP-2 and TGF-β1 treatments were applied, there was a strong increase in *Col2a1*, *Acan*, and *Snorc* mRNA levels compared with the control as early as seven days of culture. These genes were then rapidly induced and their transcription levels continuously increased with culture time. No differences were observed between normoxia and hypoxia for the same time point and same culture medium. When eUCB-MSCs were incubated with BMP-2 and TGF-β1, the mRNA expression of the specific-cartilage markers was similar to those observed in equine articular cartilage (EACs) from 14 days of culture. Therefore, eUCB-MSCs showed a very high potential for stage-specific chondrogenesis when cultured in 3D scaffolds with BMP-2 and TGF-β1 and expressed cartilage markers found in EACs.

**Figure 7:**
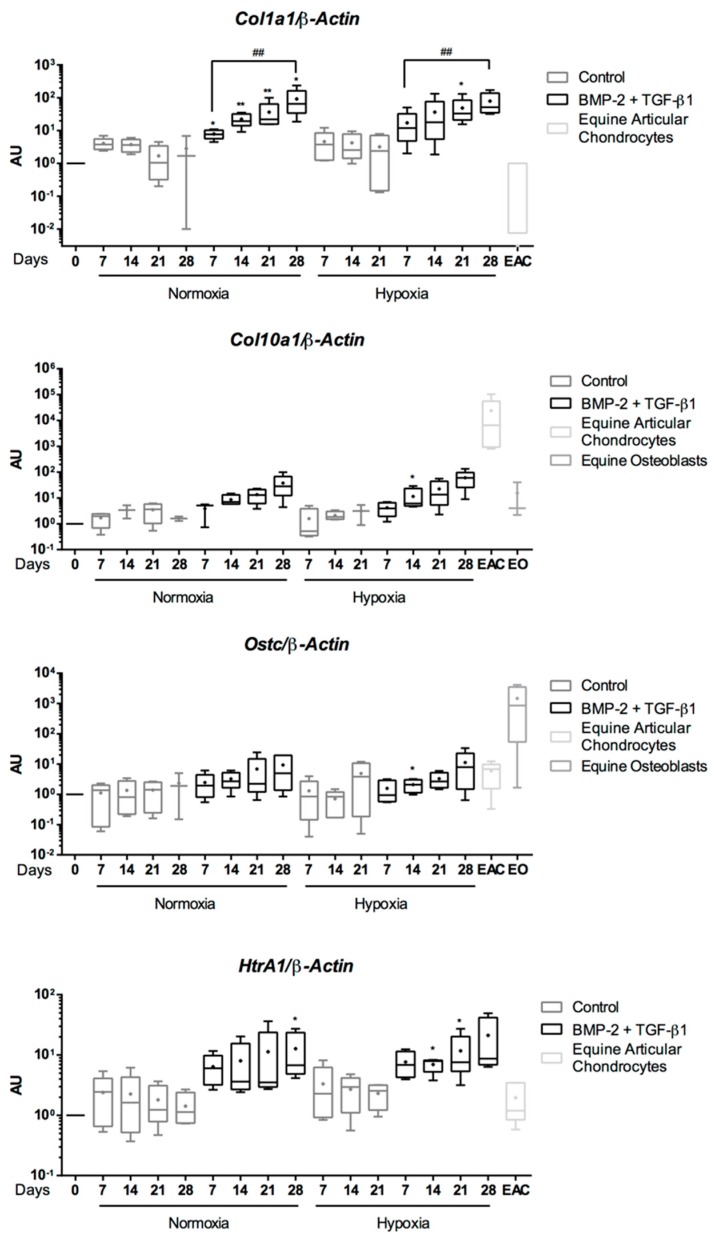

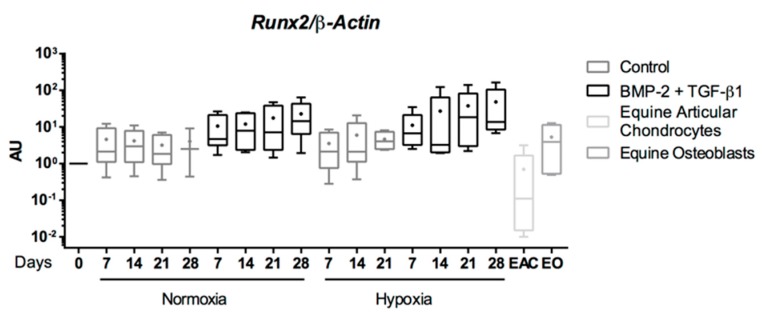
Effect of growth factors and oxygen tension on the gene expression of nonspecific-cartilage markers during the chondrogenic differentiation of equine umbilical cord blood-derived mesenchymal stem cells (eUCB-MSCs). eUCB-MSCs were cultured in type I/III collagen sponges for seven, 14, 21, and 28 days in normoxia or in hypoxia, in the absence (control), or in the presence of 50 ng/mL of BMP-2 and 10 ng/mL of TGF-β1 (BMP-2 + TGF-β1). RT-qPCR analysis of relative mRNA expression of the nonspecific-cartilage gene (*Col1a1*), of hypertrophic-cartilage markers (*Col10a1* and *Runx2*), of *HtrA1*, and of a specific-bone gene (*Ostc*) are shown. Gene expression was normalized to the *β-Actin* mRNA expression, compared with eUCB-MSCs cultured in monolayer at the fourth passage (Day 0). Equine articular chondrocyte (EAC) and equine osteoblast (EO) mRNAs were used as a positive control. Box plots represent five independent experiments performed in triplicate. Statistically significant differences between time points with the same culture medium were determined using the Friedman test (^##^ *p* < 0.01). Statistically significant differences between the culture medium at the same time point were determined using the two-tailed Mann–Whitney test (* *p* < 0.05, ** *p* < 0.01). Comparison of the differences between normoxia and hypoxia was performed using the two-tailed Mann–Whitney test, and no statistical significant differences were observed. AU: arbitrary units.

Type I collagen, considered as a fibrocartilage/dedifferentiated chondrocyte marker, is constitutively expressed in undifferentiated eUCB-MSCs. In the absence of growth factors, the mRNA level of *Col1a1* and *HtrA1* did not vary during the culture period compared with undifferentiated eUCB-MSCs, and they were similar to those of EACs (Figure 7). During the induction of chondrogenesis with BMP-2 and TGF-β1, mRNA levels of *Col1a1* and *HtrA1* increased compared with the control medium. After 28 days, mRNA levels of *Col1a1* were higher than those after seven days of induction of chondrogenesis for both oxygen conditions. To determine if culture conditions induce eUCB-MSCs hypertrophy or bone maturation, *Col10a1*, *Runx2*, and *Ostc* mRNA levels were assessed. The mRNA level of these genes did not vary in the absence of BMP-2 and TGF-β1 or in normoxia or hypoxia. In the presence of both growth factors, an increase in mRNA levels was observed between seven and 28 days, but there were no significant differences at individual time points between the absence and presence of the growth factors. The mRNA levels remained lower than those of EACs for *Col10A1* or than those of equine osteoblasts for *Ostc*. These results suggested that the growth factor combination does not induce hypertrophy or bone calcification.

**Figure 8:**
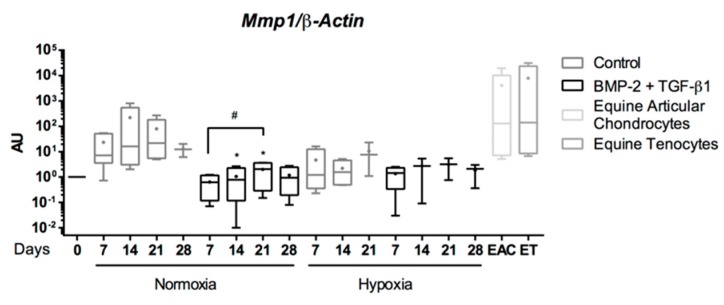

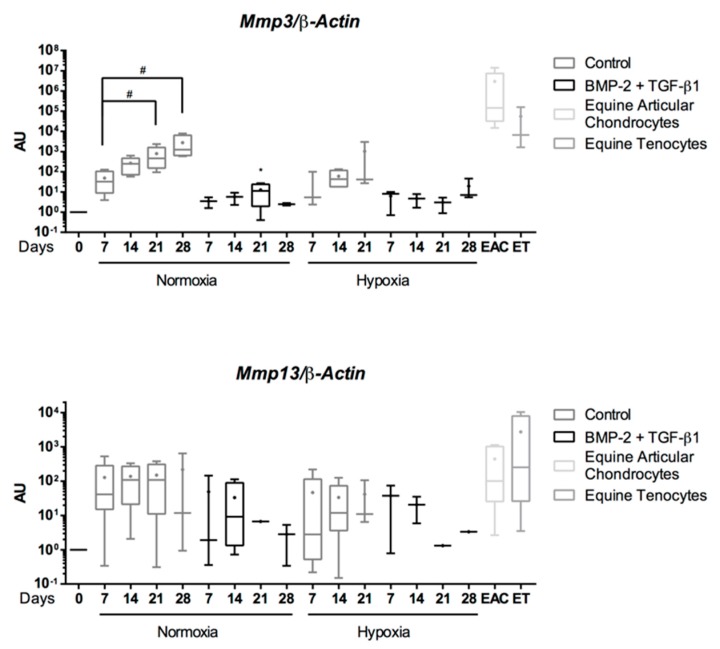
Effect of chondrogenic factors and oxygen tension on the gene expression of catabolic-cartilage markers during the chondrogenic differentiation of equine umbilical cord blood-derived mesenchymal stem cells (eUCB-MSCs). eUCB-MSCs were cultured in type I/III collagen sponges for seven, 14, 21, and 28 days in normoxia or in hypoxia, in the absence (control), or in the presence of 50 ng/mL of BMP-2 and 10 ng/mL of TGF-β1 (BMP-2 + TGF-β1). RT-qPCR analysis of relative mRNA expression of the indicated genes (*Mmp1*, *Mmp3*, and *Mmp13*) are shown. Gene expression was normalized to the *β-Actin* mRNA expression, compared with eUCB-MSCs cultured in monolayer at the fourth passage (Day 0). Equine articular chondrocyte (EAC) and equine tenocyte (ET, harvested from tendon) mRNAs were used as a positive control. Box plots represent five independent experiments performed in triplicate. Statistically significant differences between time points with the same culture medium were determined using the Friedman test (^#^ *p* < 0.05). Statistically significant differences between the culture medium at the same time point were determined using the two-tailed Mann–Whitney test (* *p* < 0.05). Comparison of the differences between normoxia and hypoxia was performed using the two-tailed Mann–Whitney test, and no statistical significant differences were observed. AU: arbitrary units.

When eUCB-MSCs were cultured in the presence of BMP-2 and TGF-β1, the mRNA levels of enzymes involved in cartilage degradation such as Mmp1, Mmp3 were similar to those of undifferentiated cells (D0) in both hypoxia and normoxia, and lower than those of cells cultured without BMP-2 and TGF-β1 (Control) (Figure 8). Regarding Mmp13, another protease also considered as an indicator of late hypertrophy, its expression appeared to be broadly equivalent in the different culture conditions but higher than the control. In all cases, mRNA levels of these genes were lower than levels observed in EAC and equine tenocytes, used as a second control.

The induction of chondrogenesis in eUCB-MSCs, cultured in collagen scaffolds with BMP-2 and TGF-β1, was associated with an upregulation of cartilage-specific genes at the mRNA level without hypertrophy or increase in the expression of matrix remodeling proteases (excepted for *HtrA1*). However, an increase in type I collagen levels was observed when the chondrogenic factors were used.

### 2.6. Effect of TGF-β3 in eUCB-MSCs Chondrogenesis

The effect of TGF-β3 alone or in combination with BMP-2 on eUCB-MSCs commitment in chondrocytes was assessed and compared with the BMP-2 + TGF-β1 treatment. Chondrogenic differentiation was then induced in a 3D collagen scaffold as previously described for seven to 28 days of culture under two oxygen tensions.

**Figure 9:**
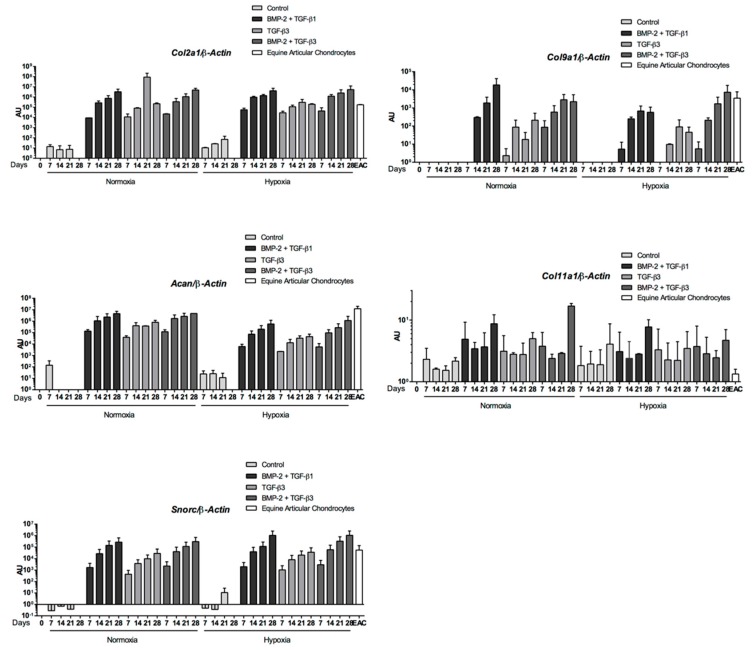
Chondrogenic gene expression during equine umbilical cord blood-derived mesenchymal stem cells (eUCB-MSCs) chondrogenesis induced by TGF-β3 ± BMP-2 compared to TGF-β1 + BMP-2. eUCB-MSCs were cultured in type I/III collagen sponges for seven, 14, 21, and 28 days in normoxia or in hypoxia, in the absence (control), or in the presence of 50 ng/mL of BMP-2 and 10 ng/mL of TGF-β1 (BMP-2 + TGF-β1) or in the presence of 10 ng/mL of TGF-β3 alone or with BMP-2 (BMP-2 + TGF-β3). RT-qPCR analysis of relative mRNA expression of the indicated genes (*Col2a1*, *Acan*, *Col9a1*, *Col11a1*, and *Snorc*) is shown. Gene expression was normalized to the *β-Actin* mRNA expression, compared with eUCB-MSCs cultured in monolayer at the fourth passage (Day 0). Equine articular chondrocyte (EAC) mRNAs were used as positive control. Histograms represent two independent experiments performed in triplicate. Statistically significant differences between time points with the same culture medium were determined using the Friedman test. Statistically significant differences between the culture medium at the same time point were determined using the Kruskal–Wallis test. Comparison of the differences between normoxia and hypoxia was performed using the two-tailed Mann–Whitney test. In each case, no statistical significant differences were observed. AU: arbitrary units.

The mRNA levels of cartilage-specific genes (*Col2a1*, *Col9a1*, *Acan*, and *Snorc*) increased only when growth factors (BMP-2 +/− TGF-β1 or β3) were added to the culture medium, regardless of oxygen conditions (Figure 9). The gene upregulation was always higher at the end of culture, reaching mRNA levels similar to those detected in EACs when cells were cultured with BMP-2 and TGF-β1 or β3. In addition, the same profiles were observed for normoxic and hypoxic conditions for the same culture medium. Compared with the control, higher levels of *Col2a1*, *Col9a1*, and *Acan* were obtained after 28 days of induction in normoxia with BMP-2 and TGF-β1. The chondrogenic commitment of eUCB-MSCs was also associated with the upregulation of the *Col11a1* gene, although the difference between conditions was not significant. This result suggests that the synthesis of cartilage matrix components was further enhanced with the combination BMP-2 + TGF-β1 or β3 compared to TGF-β3 alone. 

**Figure 10:**
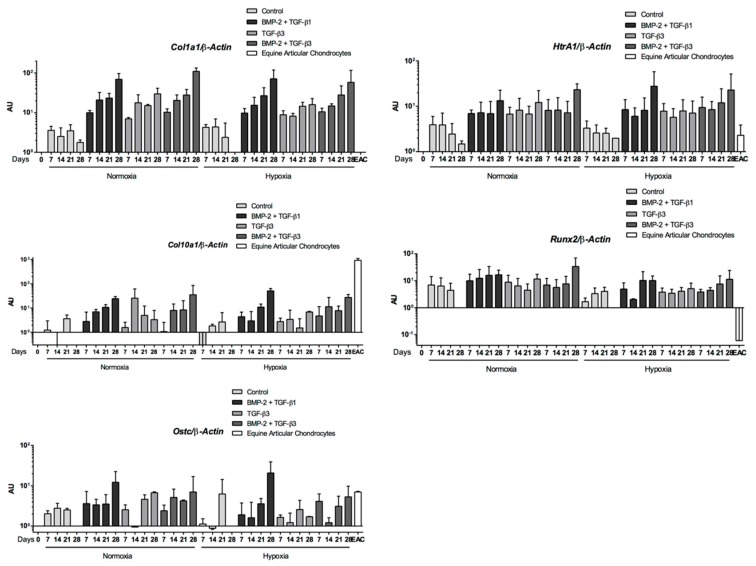
Nonspecific-cartilage gene expression during equine umbilical cord blood-derived mesenchymal stem cells (eUCB-MSCs) chondrogenesis induced by TGF-β3 ± BMP-2 compared to TGF-β1 + BMP-2. eUCB-MSCs were cultured in type I/III collagen sponges for seven, 14, 21, and 28 days in normoxia or in hypoxia, in the absence (control), or in the presence of 50 ng/mL of BMP-2 and 10 ng/mL of TGF-β1 (BMP-2 + TGF-β1), or in the presence of 10 ng/mL of TGF-β3 alone or with BMP-2 (BMP-2 + TGF-β3). Real-time RT-PCR analysis of relative mRNA expression of the noncharacteristic-cartilage genes (*Col1a1* and *HtrA1*), of hypertrophic-cartilage markers (*Col10a1*, *Runx2*, and *Alpl*), and of a specific-bone gene (*Ostc*) is shown. Gene expression was normalized to the *β-Actin* mRNA expression, compared with eUCB-MSCs cultured in monolayer at the fourth passage (Day 0). Equine articular chondrocyte (EAC) mRNAs were used as a positive control. Histograms represent two independent experiments performed in triplicate. Statistically significant differences between time points with the same culture medium were determined using the Friedman test. Statistically significant differences between the culture medium at the same time point were determined using the Kruskal–Wallis test. Comparison of the differences between normoxia and hypoxia was performed using the two-tailed Mann–Whitney test. In all cases, no statistical significant differences were detected. AU: arbitrary units.

**Figure 11:**
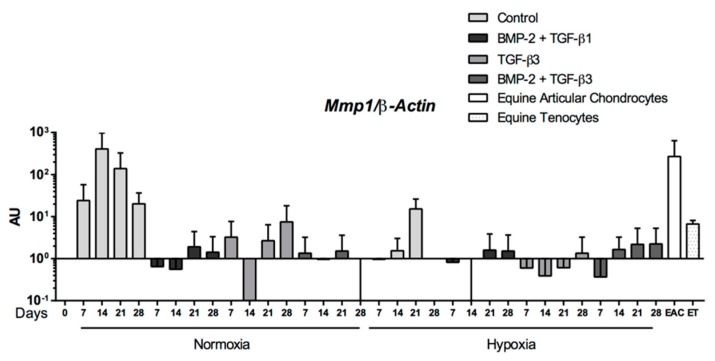

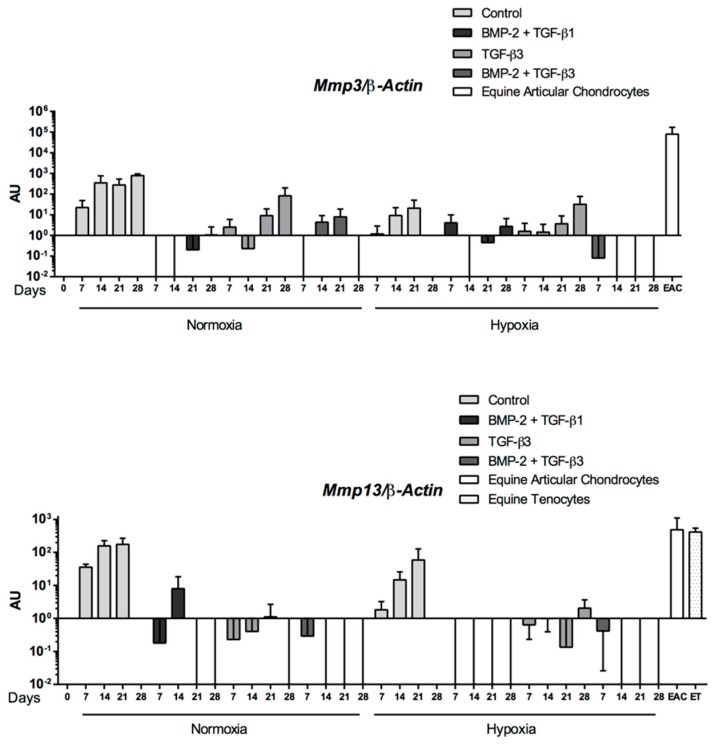
Catabolic gene expression during equine umbilical cord blood-derived mesenchymal stem cells (eUCB-MSCs) chondrogenesis induced by TGF-β3 ± BMP-2 compared to TGF-β1 + BMP-2. eUCB-MSCs were cultured in type I/III collagen sponges for seven, 14, 21, and 28 days in normoxia or in hypoxia, in the absence (control), or in the presence of 50 ng/mL of BMP-2 and 10 ng/mL of TGF-β1 (BMP-2 + TGF-β1), or in the presence of 10 ng/mL of TGF-β3 alone or with BMP-2 (BMP-2 + TGF-β3). RT-qPCR analysis of relative mRNA expression of the indicated genes (*Mmp1*, *Mmp3*, and *Mmp13*) is shown. Gene expression was normalized to the *β-Actin* mRNA expression, compared with eUCB-MSCs cultured in monolayer at the fourth passage (Day 0). Equine articular chondrocyte (EAC) and equine tenocyte (ET, harvested from tendon) mRNAs were used as a positive control. Histograms represent two independent experiments performed in triplicate. Statistically significant differences between time points with the same culture medium were determined using the Friedman test. Statistically significant differences between the culture medium at the same time point were determined using the Kruskal–Wallis test. Comparison of the differences between normoxia and hypoxia was performed using the two-tailed Mann–Whitney test. In all cases, no statistical significant differences were detected. AU: arbitrary units.

As with BMP-2 + TGF-β1, the BMP-2 + TGF-β3 combination compared with the control showed increased levels of *Col1a1* and *Htra1* mRNA as well as hypertrophic (*Col10a1*) and osteoblastic markers (*Runx2*, *Ostc*), whatever the oxygen conditions (Figure 10). However, steady-state amounts of hypertrophic or osteoblastic markers remained below that of EACs, for BMP-2 + TGF-β1 (Figure 7). Furthermore, the expression profiles of the three matrix metalloproteinases (MMPs) studied were similar for the BMP-2 + TGF-β1 and BMP-2 + TGF-β3 conditions with lower steady-state amounts than in EACs (Figure 11). TGF-β1 or TGF-β3 in combination with BMP2 enhanced chondrogenesis without appreciable matrix remodeling, hypertrophy, or osteoblastic maturation.

### 2.7. Protein Expression Analysis during eUCB-MSCs Commitment into Chondrocytes

**Figure 12:**
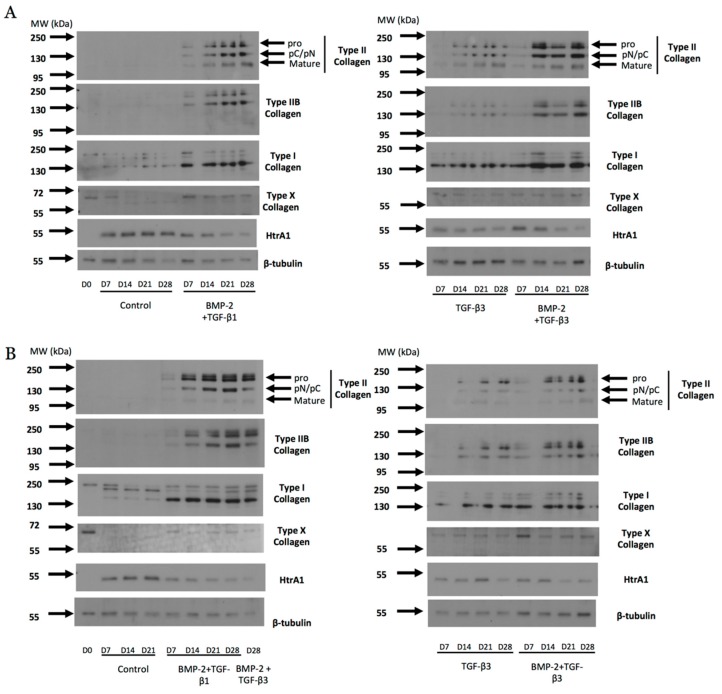
Effect of different combinations of chondrogenic factors during chondrocytes differentiation of equine umbilical cord blood-derived mesenchymal stem cells (eUCB-MSCs). eUCB-MSCs at passage four were cultured in type I/III collagen sponges for seven, 14, 21, and 28 days in normoxia (**A**) or in hypoxia (**B**), in the absence (control), or in the presence of 50 ng/mL of BMP-2 and 10 ng/mL of TGF-β1 (BMP-2 + TGF-β1), or in the presence of 10 ng/mL of TGF-β3 alone or with BMP-2 (BMP-2 + TGF-β3). Undifferentiated eUCB-MSCs were also cultured as monolayers and used as a control before differentiation (Day 0, D0). Protein extracts were analyzed in Western blots for type II, type IIB, types I and X collagens, and HtrA1 versus β-tubulin. Representative blots are shown (*n* = 5 and *n* = 2 for the culture conditions with TGF-β3 ± BMP-2 in normoxia and hypoxia, respectively). Type II collagen shows different levels of maturation forms such as type II procollagen (pro), with only C- or N-terminal propeptides (pC/pN) and the doubly cleaved form (mature form). MW: Molecular Weight, kDa: kilodaltons.

During the eUCB-MSCs chondrogenesis induced by growth factor combinations (BMP-2 + TGF-β1 or BMP-2 + TGF-β3), the expression of type II collagen was revealed in normoxia and in hypoxia (Figure 12). Type II collagen shows different forms of maturation such as type II procollagen (pro) with C- and N-terminal propeptides, procollagen with only C- or N-terminal propeptides (pC/pN), or the doubly cleaved form corresponding to the mature form. After seven days of culture in the presence of BMP-2 + TGF-β1 or TGF-β3 alone or in combination with BMP-2, the bands of type II collagen showed weak intensity which increased with time of culture, in normoxia and in hypoxia. However, in the presence of TGF-β3, the intensity was weaker compared with both combinations. The same pattern was observed with type IIB collagen, a marker of mature chondrocytes. However, the treatment with growth factors led to the overexpression of type I collagen compared with the condition without growth factors. Type X collagen expression was weakly perceptible in the presence or absence of growth factors. However, it does not seem that the eUCB-MSCs in the different experimental conditions express the 59 kDa form of type X collagen expected in denatured-reduced conditions. HtrA1 expression tended to decrease over time when BMP-2 and TGF-β1 or TGF-β3 were used.

**Figure 13:**
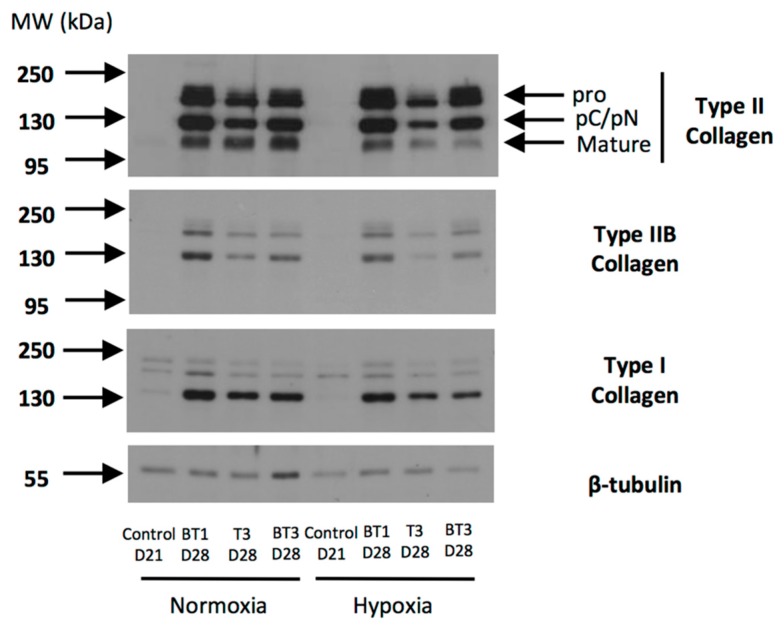
Comparison between oxygen tension and TGF-β1 or TGF-β3 in addition to BMP-2 on the protein expression of equine umbilical cord blood-derived mesenchymal stem cells (eUCB-MSCs) differentiated into chondrocytes. eUCB-MSCs at passage four were cultured in type I/III collagen sponges for 21 (D21) and 28 days (D28) in normoxia or in hypoxia, in the absence (control), or in the presence of 50 ng/mL of BMP-2 and 10 ng/mL of TGF-β1 (BT1), or 10 ng/mL of TGF-β3 (T3), or BMP-2 and TGF-β3 (BT3). Protein extracts were analyzed in Western blots for type II, type IIB, and type I versus β-tubulin. Representative blots are shown of two independent experiments. Type II collagen shows different levels of maturation forms such as type II procollagen (pro), with only C- or N-terminal propeptides (pC/pN) and the doubly cleaved form (mature form). MW: Molecular Weight, kDa: kilodaltons.

The protein expression analysis between the two oxygen tensions showed that the total type II collagen (all forms) showed higher expression in normoxia after 28 days of culture with BMP-2 and TGF-β1 than with BMP-2 and TGF-β3 (Figure 13). Furthermore, the band of the mature form of type II collagen observed at 110–120 kDa was more intense in normoxia compared with hypoxia, and with BMP-2 + TGF-β1 compared with BMP-2 + TGF-β3 in hypoxia. The same pattern was observed with type IIB collagen, although in normoxia the BMP-2 + TGF-β1 combination appeared to be more efficient for the synthesis of this marker of mature chondrocytes. Although bands of type I collagen were weak in the control, the use of chondrogenic factors led to the overexpression of this protein regardless of the conditions, with lower amounts in hypoxia compared with normoxia.

In conclusion, protein analyses confirmed that chondrogenic differentiation was induced in the presence of chondrogenic factors with a higher type II collagen content in cultures containing BMP-2 and TGF-β1 in normoxia after 28 days of culture with a more advanced maturation. However, an undesirable expression of type I collagen reduces the suitability of the newly synthesized matrix and indicates a non-stabilized phenotype of the committed cells. 

### 2.8. Gene Silencing Experiments to Overcome Type I Collagen Synthesis

The above results showed sub-optimal chondrogenic differentiation of eUCB-MSCs cultured for 28 days with BMP-2 and TGF-β1. Extending the culture period from 28 days to 42 days, we assessed the behavior of the cells committed to chondrogenic maturation and tested an RNA interference strategy.

**Figure 14:**
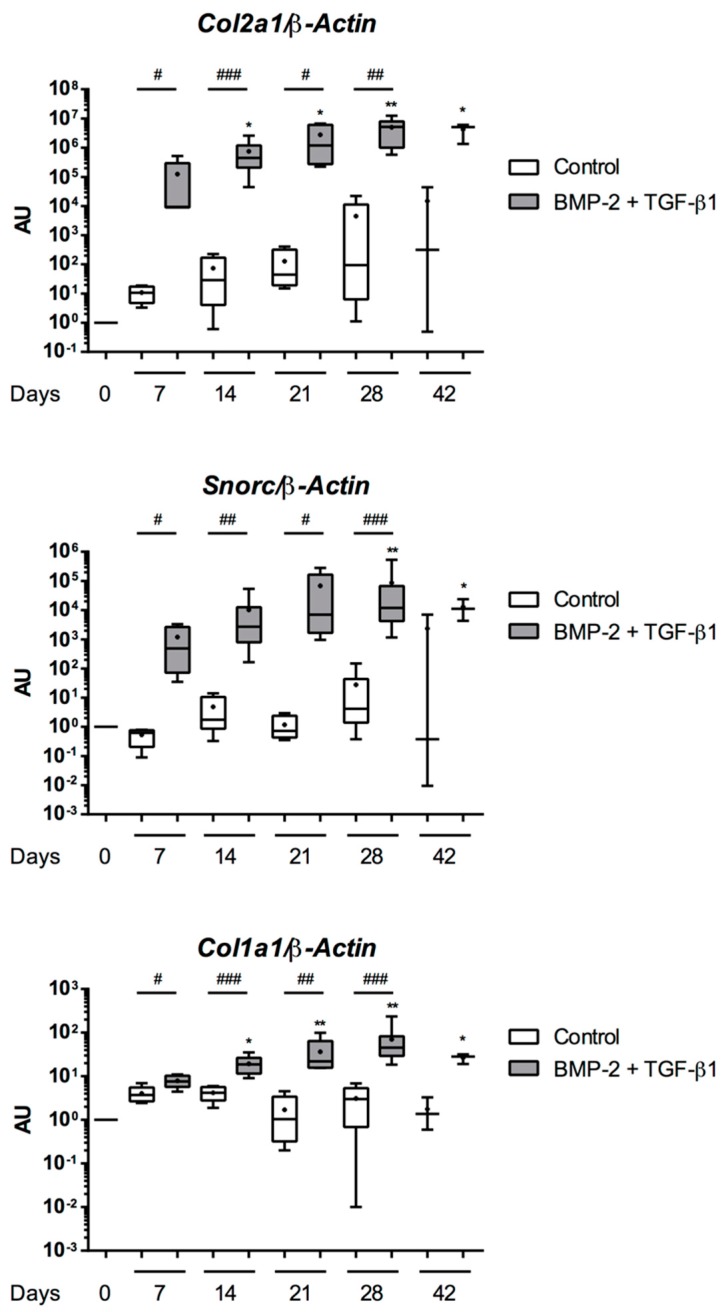
Long time culture leads to a phenotopic stabilization of equine umbilical cord blood-derived mesenchymal stem cells (eUCB-MSCs) differentiated into chondrocytes. The eUCB-MSCs were cultured in type I/III collagen sponges for seven, 14, 21, 28, or 42 days in normoxia, in the absence (control), or in the presence of 50 ng/mL of BMP-2 and 10 ng/mL of TGF-β1 (BMP-2 + TGF-β1). RT-qPCR analysis of relative mRNA expression of two cartilage-specific genes (*Col2a1* and *Snorc*) and an unusual marker of hyaline cartilage (*Col1a1*) is shown. Gene expression was normalized to the *β-Actin* mRNA expression, compared with undifferentiated eUCB-MSCs cultured in monolayer at the fourth passage (Day 0). Box plots represent at least five (and up to eight) independent experiments performed in triplicate (*n* = 3 for 42 days of culture). Statistically significant differences were determined using the two-tailed Mann–Whitney test (^#^ *p* < 0.05, ^##^ *p* < 0.01, ^###^ *p* < 0.001). Statistically significant differences between time points with the same culture medium were determined using the Friedman test (* *p* < 0.05, ** *p* < 0.01). AU: arbitrary units.

The gene expression of cartilage-specific markers such as *Col2a1* and *Snorc* was similar for 28 and 42 days of culture with BMP-2 and TGF-β1 (Figure 14). The *Col1a1* mRNA level increased until 28 days of culture and then stabilized. 

**Figure 15:**
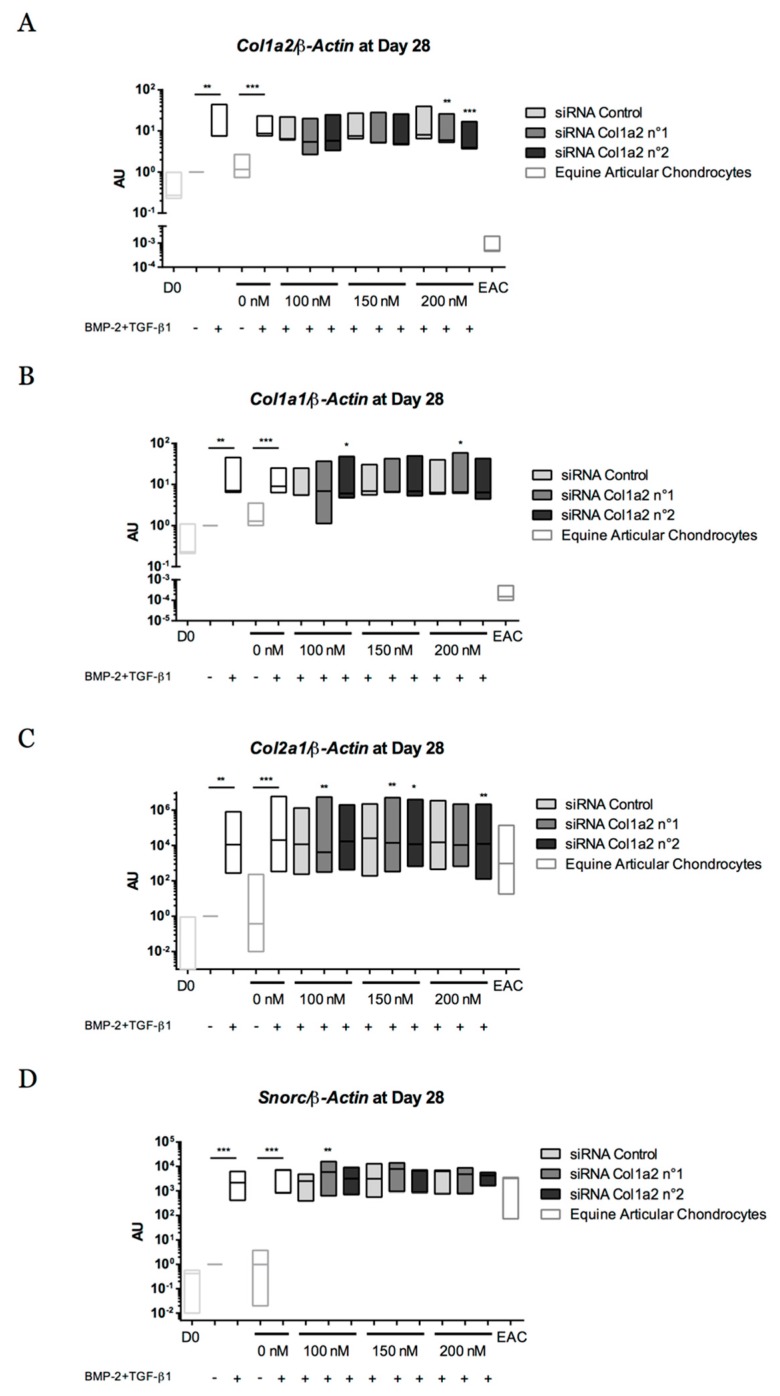
Effect of successive transfections of *Col1a2* siRNA at the transcriptional level after 28 days of chondrogenic differentiation of equine umbilical cord blood-derived mesenchymal stem cells (eUCB-MSCs). At passage four, eUCB-MSCs were cultured in type I/III collagen sponges for 28 days in normoxia, in the absence or in the presence of 50 ng/mL of BMP-2 and 10 ng/mL of TGF-β1 (BMP-2 + TGF-β1). Four transfections of two different sequences of siRNA targeting *Col1a2* at 100, 150, or 200 nM were performed between 14 and 24 days of culture. RT-qPCR analysis of relative mRNA expression of *Col1a2* (**A**), *Col1a1* (**B**), *Col2a1* (**C**), and *Snorc* (**D**) is shown. Gene expression was normalized to the *β-Actin* mRNA expression, compared with eUCB-MSCs cultured in sponges for 28 days without BMP-2 + TGF-β1. Undifferentiated eUCB-MSCs at the fourth passage were used as a control before differentiation (Day 0, D0). mRNA extracts obtained from equine articular chondrocytes (EAC) released from cartilage after overnight enzymatic digestion were used as a control. Floating bars represent three independent experiments performed in triplicate. Statistically significant differences with the case transfected with a control siRNA at the related concentration were determined using the Student’s *t*-test (* *p* < 0.05, ** *p* < 0.01, *** *p* < 0.001). AU: arbitrary units.

To overcome the expression of type I collagen, gene silencing experiments targeting *Col1a2*, encoding the α*2* chain of type I collagen, were performed with successive transfections of two siRNAs (No. 1 and No. 2) (at 14, 17, 21, 24, and 35 days) during a culture period of 28 and 42 days. During the first 28 days of chondrogenic differentiation, the two siRNAs targeting *Col1a2* mRNA used at 200 nM led to a decrease in *Col1a2* gene expression compared with the control siRNA (Figure 15A). An average decrease of 31.5% and 55% of *Col1a2* mRNA level was observed with siRNA No. 1 and with siRNA No. 2, respectively, compared with the control siRNA. Targeting *Col1a2* mRNA did not seem to modify *Col1a1* expression, although siRNA No. 1 at 200 nM and siRNA No. 2 at 100 nM induced a slight increase in *Col1a1* mRNA levels (Figure 15B). Regarding the expression of the cartilage-specific genes *Col2a1* and *Snorc*, the two siRNAs did not show deleterious effects on their expression level except siRNA No. 2 at 200 nM on the *Col2a1* (but not *Snorc*) mRNA level, for which a slight decrease was observed (Figure 15C,D).

**Figure 16:**
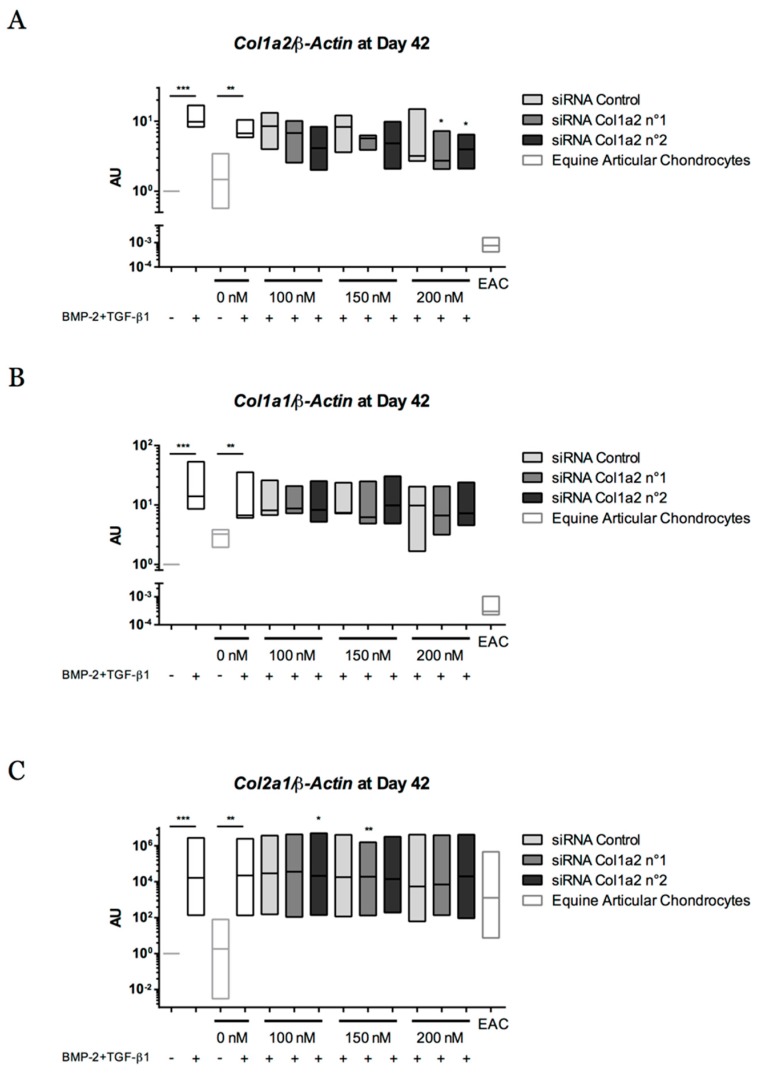

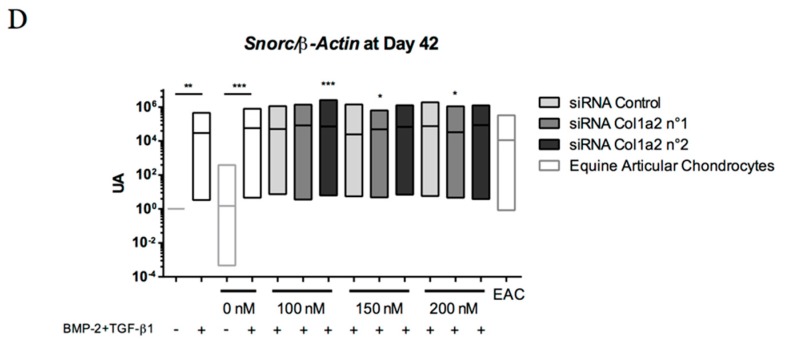
Effect of successive transfections of *Col1a2* siRNA at the transcriptional level after 42 days of chondrogenic differentiation of equine umbilical cord blood-derived mesenchymal stem cells (eUCB-MSCs). At passage four, eUCB-MSCs were cultured in type I/III collagen sponges for 42 days in normoxia, in the absence or in the presence of 50 ng/mL of BMP-2 and 10 ng/mL of TGF-β1 (BMP-2 + TGF-β1). Five transfections of two different sequences of siRNA targeting *Col1a2* at 100, 150, or 200 nM were performed between 14 and 24 days and at 35 days of culture. Real-time RT-PCR analysis of relative mRNA expression of *Col1a2* (**A**), *Col1a1* (**B**), *Col2a1* (**C**), and *Snorc* (**D**) is shown. Gene expression was normalized to the *β-Actin* mRNA expression, compared with eUCB-MSCs cultured in sponges for 42 days without BMP-2 + TGF-β1. The mRNA extracts obtained from equine articular chondrocytes (EAC) released from cartilage after overnight enzymatic digestion were used as a control. Floating bars represent three independent experiments performed in triplicate. Statistically significant differences with the case transfected with a control siRNA at the related concentration were determined using the Student’s *t*-test (* *p* < 0.05, ** *p* < 0.01, *** *p* < 0.001). AU: arbitrary units.

When the culture period was extended to 42 days, a decrease of 42.2% and 39.8% in *Col1a2* mRNA levels compared with the control siRNA was observed for siRNA No. 1 and siRNA No. 2 (200 nM), respectively (Figure 16A). This concentration of 200 nM did not induce variation in *Col1a1* and *Col2a1* mRNA levels (Figure 16B,C). siRNA No. 1 was associated with a slight decrease in the transcriptional expression of *Snorc* (Figure 16D).

**Figure 17:**
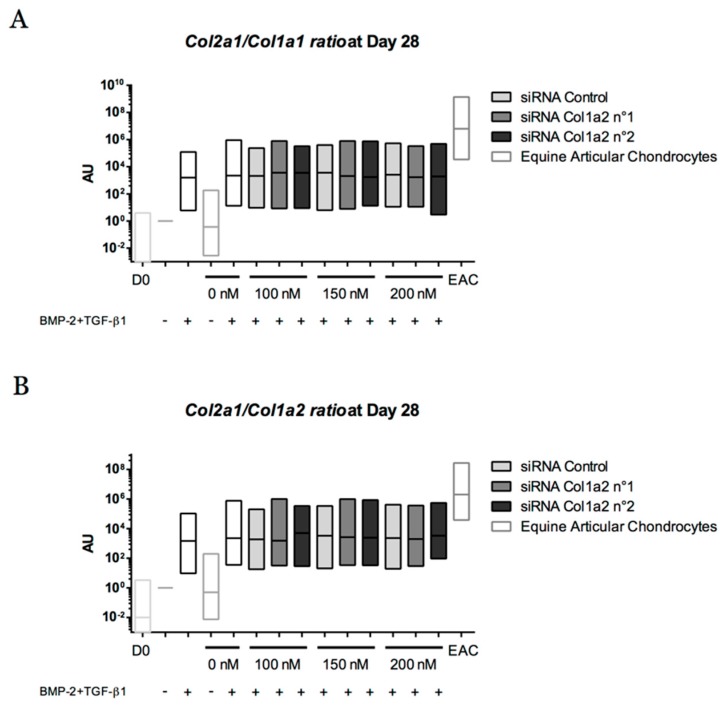

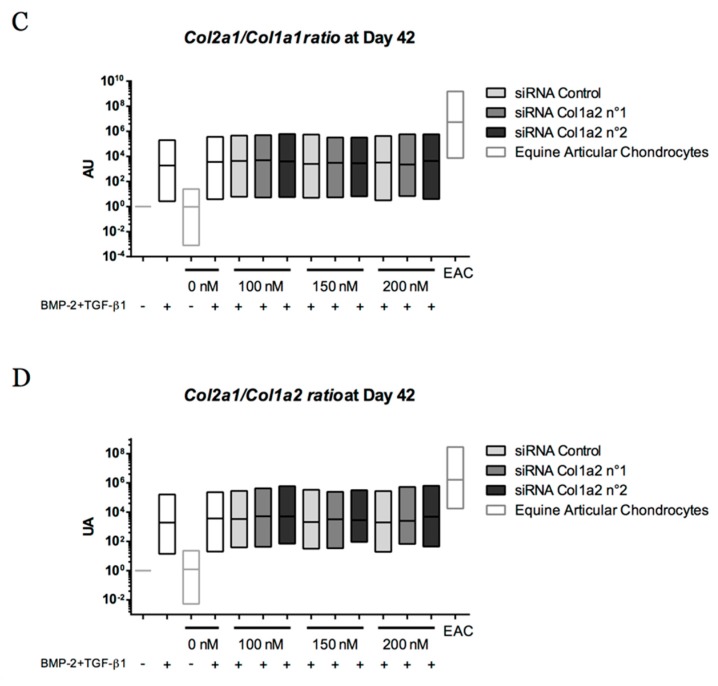
Effect of successive transfections of *Col1a2* siRNA on the *Col2a1* mRNA/*Col1a1* mRNA and *Col2a1* mRNA/*Col1a2* mRNA ratios during 28 and 42 days of chondrogenic differentiation of equine umbilical cord blood-derived mesenchymal stem cells (eUCB-MSCs). At passage four, eUCB-MSCs were incubated in the same experimental conditions as those described in the legends of Figure 15 and Figure 16, and gene expression analysis was performed in an identical manner. The functional index of the eUCB-MSCs differentiated into chondrocytes was determined with the ratio of *Col2a1* mRNA:*Col1a1* mRNA after 28 days of culture (**A**), *Col2a1* mRNA:*Col1a2* mRNA after 28 days of culture (**B**), *Col2a1* mRNA:*Col1a1* mRNA after 42 days of culture (**C**), and *Col2a1* mRNA:*Col1a2* mRNA after 42 days of culture (**D**). Undifferentiated eUCB-MSCs at the fourth passage were used as a control before differentiation (Day 0, D0). mRNA extracts obtained from equine articular chondrocytes (EAC) released from cartilage after overnight enzymatic digestion were used as a control. Floating bars represent three independent experiments performed in triplicate. Statistically significant differences with the case transfected with a control siRNA at the related concentration were determined using the Student’s *t*-test. No statistical significant differences were observed. AU: arbitrary units.

To better understand the phenotype obtained after chondrogenic differentiation of eUCB-MSCs, the functional differentiation index was calculated (Figure 17). It corresponds to the ratio of *Col2a1* mRNA to *Col1a1* or *Col1a2* mRNA levels after 28 and 42 days. The chondrogenic factors increased (i) the *Col2a1:Col1a1* ratios by 41,000 (after 28 days) and 66,000 (after 42 days) fold, and (ii) the *Col2a1:Col1a2* ratios by 35,000 (after 28 days) and 55,000 (after 42 days) compared with the control. Moreover, they approached—but did not attain—levels observed in EACs. Gene silencing targeting *Col1a2* mRNA did not improve the ratio, regardless of the conditions. 

## 3. Discussion

MSCs are increasingly studied for their potential in tissue engineering. The horse is an ideal animal model for the study of the musculoskeletal system, but extensive knowledge of equine MSCs is required for future clinical applications in veterinary medicine. In this study, we isolated and characterized equine UCB-MSCs. We determined their potential for cartilage tissue engineering using type I/III collagen sponges and chondrogenic factors under two oxygen conditions to assess the optimal conditions for chondrogenic differentiation.

Equine UCB is a source of MSCs with a painless and non-invasive harvesting procedure. The success rate in MSCs isolation was 100% when eUCB samples were treated within 40 h after foaling. Lower success rates (57%) are reported in the scientific literature, despite faster treatment times (a mean of 15 h) [40]. Improved culture methods that separate red and white blood cells have a success rate of 100% [41]. In humans, successful derivation of MSCs from UCB ranges from 29 to 90% [42,43]. The difference observed between horses and humans may be due to sample volume, collection methods, or intrinsic cellular properties.

Regenerative medicine based on MSCs require high cell numbers. The first eUCB-MSCs appear nine days after seeding (P0), as described previously [17]. These cells multiplied rapidly and proliferative activity remained high until P15. When FGF-2 was added to the culture medium during the eUCB-MSCs expansion stage, there was an increase in population doublings. A comparative study of expansion in human adult bone marrow-derived MSCs with or without FGF-2 showed that FGF-2 extends the level of expansion, reaching target cell numbers more rapidly [44]. However, our results show that supplementation with FGF-2 does not prevent replicative senescence after P15.

Previous studies have shown that MSCs have a limited life span after several cell divisions associated with replicative senescence [45]. However, assessing mRNA expression changes indicative of in vitro senescence of MSCs revealed no variation at the mRNA level, except for p21, involved in cell cycle arrest, which increased after P15. Our results suggest that cells must be used before P15 to avoid the risk of cell senescence.

The multipotency of MSCs is based on their ability to differentiate into cells of the mesodermal lineage [46]. Studies have reported a difference in MSCs differentiation capacity according to their provenance. In humans, UCB-MSCs show no adipocyte differentiation in contrast to cells derived from bone marrow and adipose tissue [19]. Although the time required for chondrocyte differentiation is identical for these three sources of MSCs in the horse, differentiation into osteoblasts and adipocytes takes longer for eUCB-MSCs compared to other sources [47]. In this study, the eUCB-MSCs differentiated easily into osteoblasts and chondrocytes, but they failed to differentiate into adipocytes. Reports have demonstrated adipogenic differentiation of eUCB-MSCs only when 15% rabbit serum was used in the differentiation medium [17,40]; therefore, the protocol may probably need to be modified to demonstrate the tripotency of eUCB-MSCs. 

The eUCB-MSCs were characterized by immunophenotyping focusing on ISCT-recommended markers (CD73, CD90, and CD105) [15] and other MSCs markers well-described in the literature (CD29 and CD44) [16]. Our results show that CD29, CD44, and CD90 are expressed by eUCB-MSCs at P4. However, for eUCB-MSCs, only a part of the cell population expressed CD73, as described by De Schauwer et al. [48]. eUCB-MSCs were negative for CD105 in our study. Recently, canine MSCs isolated from adipose tissue were also reported to be CD105^−^ negative [49]. For equine MSCs derived from adipose tissue, CD105 flow cytometry analysis using the same clone as the one used in this study showed a strong positive signal [50]. This variation in CD105 expression may be due to cell origins or to the enzymes used for subculturing cells, as it was demonstrated for CD14 in equine MSCs [51].

This study aimed to determine eUCB-MSCs potential for cartilage tissue engineering using type I/III collagen sponges as scaffolds. All of our results showed that these cells have the capacity to synthesize a specific protein representative of hyaline articular cartilage in collagen sponges in the presence of chondrogenic factors. The comparison with other studies is not straightforward because other protocols perform chondrocyte differentiation in monolayer cultures [52], in pellet cultures [50], or in hydrogels [53] in the presence of only one growth factor. Equine MSCs derived from adipose tissue cultured in a micromass system at P4 showed only a non-significant induction of type II collagen and aggrecan [50] and at P2, Alcian blue staining was more intense at the periphery of cell pellets, giving evidence of an incomplete and heterogenous differentiation. Although pellet cultures promote cell-cell interactions and close contacts with the matrix which are essential for chondrogenic commitment at the condensation stage of endochondral ossification, this 3D model limits oxygen and nutrient diffusion. Collagen sponge scaffolds, in contrast to micromass cultures, are porous, allowing seeded cells to migrate and providing efficient nutrient and gas diffusion. Moreover, this culture model limits the cell-cell interactions which are not observed in the resting superficial zone of articular cartilage. The 3D scaffolds have already been used for chondrocyte differentiation with equine MSCs and lead to the synthesis of a neocartilage more similar to hyaline cartilage compared than that produced in pellet cultures [54]. However, there are no differences in *Col2a1* mRNA levels between the culture models and the authors did not carry out comparisons with undifferentiated MSCs or with equine cartilage as a gold standard as in the present study.

Previous chondrogenic differentiation experiments carried out on equine MSCs employed a unique member of the TGF-β family. For example, in bone marrow-derived MSCs, *Acan* and *Col2a1* upregulation was demonstrated in hypoxia after 14 and 21 days of culture with TGF-β3 [55]. This growth factor was the main one used in various protocols, for which culture time generally does not exceed 21 days [45,54,56]. In some studies, TGF-β1 was also used and showed increased expression of type II collagen and aggrecan in eUCB-MSCs [23]. In our study and for the first time, the effect of culturing eUCB-MSCs in the absence or presence of BMP-2 (50 ng/mL) combining with TGF-β1 or TGF-β3 (10 ng/mL) in type I/III collagen sponges was evaluated based on the expression of several markers, specific to or non-specific to hyaline cartilage during culture for up to 42 days. The expression of *Col2a1*, *Acan*, and *Snorc* occurred only in the presence of chondrogenic factors and increased gradually with time, in normoxia and in hypoxia. After 28 days of chondrogenic differentiation, the combination of BMP-2 and TGF-β1 showed higher expression of type II collagen at the protein level than the BMP-2 + TGF-β3 combination. Furthermore, mRNA levels of *Col9a1* were higher when BMP-2 and TGF-β1 were used.

As studies on 3D cartilage tissue engineering using human MSCs have highlighted the risk of cell hypertrophy in newly synthesized tissue [31], several markers of hypertrophic chondrocytes, non-specific to cartilage or osteoblasts, were assessed. However, in our equine MSC model, at the mRNA and the protein level, type X collagen, the main marker of hypertrophy and osteoarthritis, was not expressed or only weakly expressed, as was Mmp13, the main protease involved in cartilage degradation upregulated in late hypertrophic chondrocytes. Furthermore, HtrA1, a serine protease that can degrade a variety of extracellular matrix (collagens and aggrecans) as well as TGF-ß/BMPs receptors, was detected at the protein level in the absence of growth factors, whereas in the presence of BMP-2 and TGF-β1, HtrA1 was downregulated gradually over time. Overall, our results suggest that our culture model mainly promotes the differentiation of equine MSCs into chondrocytes without hypertrophy.

Previous studies have demonstrated the importance of reduced oxygen tension to enhance cartilage matrix formation in equine MSCs [54] and to inhibit the type I collagen expression of dedifferentiated chondrocytes [57]. Culturing MSCs under 5% oxygen tension results in higher glycosaminoglycan and type II collagen levels than cultures performed under 21% oxygen normoxia. Moreover, an upregulation of chondrogenesis-related genes is observed with equine bone marrow-derived MSCs expanded in hypoxia [55]. The influence of hypoxia (2% O_2_) for chondrogenesis with human adipose tissue-derived MSCs has been studied in pellet cultures in the presence of BMP-2 and TGF-β3. The results suggest that hypoxia promotes the maintenance of a chondrocyte phenotype at the mRNA level, whereas MSCs cultured in normoxia (21% O_2_) had a hypertrophic phenotype with an upregulation in the gene expression of *COL10A1* and *MMP13* [28]. In this study, no evidence of a hypertrophic phenotype was observed when the chondrogenic differentiation was performed in normoxia. Furthermore, although hypoxia promotes the high expression of all forms of type II collagen, the mature alpha1(II) chain was more expressed in normoxia. However, the expression level of type I collagen is higher in normoxia compared to hypoxia. A strategy consisting in alternating the oxic condition (normoxia first and hypoxia for the culture ending) could improve the phenotype of committed MSCs into chondrocytes.

Although TGF-β1 treatment acts to stimulate all types of collagen synthesis [58], previous reports on cartilage engineering with MSCs on the equine model did not explore type I collagen expression. Type I collagen is a basal marker of undifferentiated MSCs, but our results showed that the use of growth factors to promote chondrogenesis led to an upregulation of this marker. Even though our results with long culture times suggested that the transcriptional expression of type I collagen stabilized between 28 and 42 days of culture, we performed a gene silencing experiment to attempt to reduce type I collagen gene expression. Preliminary experiments using siRNA targeting *Col1a1* mRNA were performed and the concentrations tested ranged between 5 and 200 nM (data not shown). Only concentrations above 100 nM appeared to induce a decrease of *Col1a1* mRNA, but the results varied with the MSC strain. Because type I collagen is composed of two times more α1 than α2 chains, we targeted a decrease in the synthesis of the α2 chains to provoke better intracellular degradation. Thus, two sequences of siRNA targeting *Col1a2* mRNA were assessed with encouraging results at the transcriptional level. A maximum decrease of 55% on the targeting gene was observed after 28 days of culture with siRNA No. 2 at 200 nM. This siRNA sequence led to a decrease of only 39.8% after 42 days of culture. As it seems that this interference strategy is not successful at all in our conditions for eUCB-MSCs, alternative strategies to counteract collagen type I expression could be tested, such as the use of miRNAs or anti-miRNAs, or even culture of the cells in bioreactors. In mouse MSCs, overexpression of miRNA 145 decreases Sox9 expression, and culture without growth factors with anti-miR-145 induces the expression of chondrogenic markers (*Col2a1*, *Acan*, *Comp*, *Col9a2*, and *Col11a1*) [59]. 

Here we characterized equine UCB-MSCs; they showed great potential for cartilage tissue engineering when seeded in type I/III collagen sponges in the presence of BMP-2 and TGF-β1 for 28 and 42 days of culture. However, further extensive studies are needed to determine if oxygen tension improves the quality of neo-synthesized cartilage by reducing type I collagen expression. Nonetheless, these MSCs possess properties of expansion allow banking and can be a reliable source for cartilage repair/regeneration. This study provides new insights for the development of future strategies in veterinary medicine.

## 4. Material and Methods

### 4.1. eUCB-MSCs Isolation and Culture

The eUCB was collected from 24 foals immediately after foaling by venipuncture of the umbilical veins performed with a 16G hypodermic needle attached to a 250-mL blood transfusion collection bag (MSE3500Q, Macopharma , Mouvaux, France) containing 35 mL of citrate phosphate-dextrose-adenine as the anticoagulant solution. Twenty-four eUCB samples were collected from *Ecuries Lebourgeois* (La Louverie, Semallé, France) and *Haras de Sou* (Bursard, France). The eUCB samples were processed within 9 to 62 h after collection. To isolate mononuclear cells (MNCs), each UCB sample was diluted 1:1 with phosphate-buffered saline (PBS) and carefully mixed with Ficoll-Paque PREMIUM (GE Healthcare Bio-Sciences, Chicago, IL, USA). After density gradient centrifugation at 400 g for 30 min at room temperature (RT), MNCs were washed with PBS by centrifugation at 400 g for 10 min at RT. Pellets were suspended in isolation medium consisting of low glucose-Dulbecco’s Modified Eagle Medium (LG-DMEM, Life Technologies, Carlsbad, CA, USA) containing 30% Fetal Calf Serum (FCS, Life Technologies) and 10^−7^ M dexamethasone (Sigma-Aldrich, Saint Louis, MO, USA). Cells were seeded in culture flasks and incubated at 37 °C in a 5% CO_2_ humidified atmosphere. A mix of antimicrobials composed of 100 IU/mL of penicillin (Pan Pharma, Luitré, France), 100 µg/mL of erythromycin (Amdipharm, London, UK), and 0.25 mg/mL of fungizone (Bristol-Myers Squibb, New York City, NY, USA) was added to all of the culture media used in this study. Non-adherent cells were removed 24 h after initial plating. The medium was changed twice weekly until adherent cells appeared, defined as passage zero (P0). After the appearance of several colonies in 22 samples, cells were detached using trypsin/EDTA (Life Technologies) and then re-seeded at 5000 cells/cm^2^ (passage one, P1), and so on until confluence reached 80% at passage 20 (P20). Cell expansion was performed in LG-DMEM containing 20% FCS. Digital images were obtained to document cell morphology at passage zero and two using a phase contrast microscopy and Zen software (Zeiss, Oberkochen, Germany).

Equine articular chondrocytes (EAC) were prepared from macroscopically healthy zones of cartilage in metacarpophalangeal joint in horses undergoing euthanasia at the Centre d’Imagerie et de Recherche sur les Affections Locomotrices Equines (CIRALE, Goustranville, France). Cartilage samples were cut into small slices, then chondrocytes were isolated by sequential digestion for 45 min at 37 °C with 2 mg/mL of type XIV protease (Sigma-Aldrich, St. Louis, MO, USA), and then overnight at 37 °C with 1 mg/mL of type I collagenase (from *Clostridium histolyticum*, Invitrogen Life Technologies, Carlsbad, CA, USA), as previously described in a human model [60]. The cell suspension was filtered through a 70-µm mesh nylon membrane and centrifuged at 200 g for 10 min. The pellets were resuspended in Trizol (Invitrogen Life Technologies, Carlsbad, CA, USA) and RNA extraction was carried out according to the manufacturer’s protocol. For Western blot analysis, cartilage slices were ground in liquid nitrogen and protein extraction was performed with RIPA-lysis buffer. EAC extracts were used in real-time reverse transcription-polymerase chain reaction (RT-PCR) and Western blot as controls. mRNA extracts from equine tenocytes harvested from tendon were used in RT-PCR as controls.

### 4.2. Proliferation Capacity and Senescence Analysis

eUCB-MSCs were cultured in expansion medium with LG-DMEM, 20% FCS ± 5 ng/mL FGF-2 (Miltenyi Biotec, Bergisch Gladbach, Germany). Every seven days, the population doubling level was calculated for each passage (from P1 to P20) as follows: population doublings (PDs) = (log10(NH) − log10(NI))/log10(2), where NI is the number of seeded cells and NH is the number of harvested cells. For the cumulative population doubling, the PD was added to all PDs of previous passages. For each passage and medium condition, cells were harvested for RNA analysis.

### 4.3. Immunophenotyping

At passage four, eUCB-MSCs were analyzed by flow cytometry using a Gallios flow cytometer (Beckman Coulter, Brea, CA, USA) with a panel of antibodies (Table 1). Flow-check Pro Fluorospheres (Beckman Coulter, Brea, CA, USA) were used to verify instrument optical alignment and fluidics. Flow-Set Pro Fluorospheres (Beckman Coulter, Brea, CA, USA) were used to verify instrument sensibility. eUCB-MSCs were harvested, washed, and resuspended in PBS at a density of 10^6^ cells/mL. Cell suspensions were incubated with monoclonal antibodies for 30 min at 4 °C in the dark, followed by a wash and, for CD73 and CD90, a secondary antibody incubation at 4 °C for 30 min in the dark. Subsequently, the cells were washed and resuspended in 500 µL of PBS. All primary and secondary antibodies are listed in Table 1. The respective mouse isotype antibodies served as controls. A minimum of 20,000 events were acquired for each antibody using Gallios software (Beckman Coulter, Brea, CA, USA) and analyzed with FlowJo software (Tree Star, Ashland, OR, USA).

### 4.4. Multilineage Capacity

The capacity of eUCB-MSCs to differentiate in osteogenic, adipogenic, and chondrogenic lineages was performed at P4. Cells were seeded in six-well plates and grown until 50% confluency. To induce osteogenesis, cells were cultured for 21 days in osteogenic medium containing LG-DMEM, 10% FCS, 10^−7^ M dexamethasone, 100 µM ascorbic acid-2-phosphate (Sigma-Aldrich, St. Louis, MO, USA), and 10 mM β-glycerophosphate (Sigma-Aldrich, St. Louis, MO, USA), and the culture medium was changed once a week. After fixation with 10% formalin solution (Sigma-Aldrich, St. Louis, MO, USA) for 10 min at RT, osteogenic differentiation was assessed by Alizarin Red S staining for calcium deposition.

To induce adipogenesis, cells were cultured with three cycles of adipogenic medium (for 72 h) and maintenance medium (24 h). The adipogenic medium consisted of LG-DMEM, 10% FCS, 10^−6^ M dexamethasone, 0.5 mM 3-isobutyl-1-methyl-xanthine (Sigma-Aldrich, St. Louis, MO, USA), 0.2 nM indomethacin (Sigma-Aldrich, St. Louis, MO, USA), and 10 µg/mL recombinant human insulin (Sigma Aldrich, St. Louis, MO, USA). The maintenance medium consisted of LG-DMEM, 10% FCS, and 10 µg/mL recombinant human insulin. After these three cycles of culture, cells were incubated for seven days in maintenance medium and fixed as previously described. Evaluation of positive adipogenesis was done by Oil Red O staining to observe lipid droplets.

To induce chondrogenesis, cells were cultured with incomplete chondrogenic medium (ICM) for 14 days and changed twice a week. ICM is composed of high glucose-DMEM (HG-DMEM, 4.5 g/L) supplemented with 50 µg/mL ascorbic acid-2-phosphate, 100 µg/mL sodium pyruvate (Invitrogen Life Technologies, Carlsbad, CA, USA), 40 µg/mL proline (Sigma-Aldrich, St. Louis, MO, USA), 1:100 dilution of insulin transferrin selenium (TermoFisher Scientific, Waltham, MA, USA), and 10^−7^ M dexamethasone. Two growth factors were added; 50 ng/mL of bone morphogenetic protein-2 (rhBMP-2, inductOs to Wyeth Europa Ltd., Maidenhead, UK) and 10 ng/mL of rhTGF-β1 (Miltenyi Biotec, Bergisch Gladbach, Germany). After the fixation of cells as previously described, chondrogenesis was evaluated using Alcian Blue staining to observe acidic polysaccharides such as glycosaminoglycans. 

In parallel of each differentiation assay, an equal number of cells were maintained in the MSC expansion medium as a control.

### 4.5. Chondrogenic Differentiation in 3D Scaffolds

The scaffold was manufactured by Symatèse Biomatériaux (Chaponost, France). These collagen sponges are 2 mm in thickness, 5 mm in diameter, with a 100 µm pore size. They are composed of native type I (90 to 95%) and type III (5 to 10%) collagens from calf skin. Collagens are crosslinked with glutaraldehyde to increase their stability. They are sterilized with β-radiation.

eUCB-MSCs were subcultured as monolayers until passage three, trypsinized, and suspended in ICM. Cells were seeded onto the sponges (5 × 10^5^ cells/sponges) in a 96-well culture plates which were then incubated at 37 °C under 5% CO_2_. After 1 h, sponges were transferred to 24-well plates with ICM in the presence or absence of 50 ng/mL rhBMP-2 and 10 ng/mL rhTGF-β1 or 10 ng/mL rhTGF-β3 (Bio-Techne, Minneapolis, Minn, USA) ± 50 ng/mL rhBMP-2. Culture times were seven, 14, 14, 21, 28, or 42 days at 37 °C under 5% CO_2_ in normoxia (21% O_2_) or in hypoxia (<3% O_2_), and the medium was changed twice a week. Sponges were harvested for RNA and proteins were analyzed at each time point and for each culture medium condition. eUCB-MSCs monolayers cultured with expansion medium were used as a control (day zero, D0).

### 4.6. Gene Silencing Experiments

After three passages, eUCB-MSCs were harvested by trypsinization and seeded onto collagen sponges as previously described. Seeded sponges were cultured with ICM in the presence of 50 ng/mL rhBMP-2 and 10 ng/mL rhTGF-β1 for 28 or 42 days under 5% CO_2_ in normoxia. The medium was changed twice a week. At day 14, 17, 21, 24, and 35, eUCB-MSCs were transfected with a mix of INTERFERin™ (Polyplus Transfection SA, Illkirch-Graffenstaden, France), OptiMEM (Invitrogen Life Technologies, Carlsbad, CA, USA) and small interfering RNA (siRNA) at 200 nM according to the manufacturer’s instructions. siRNAs specifically targeted the *Col1a2* mRNA (sequence of siRNA n°1: 5′-GAUGGCUGCUCUAGAAAGA-3′, sequence of siRNA n°2: 5′-GCCAAGAACUGGUACAGAA-3′, Eurogentec, Liège, Belgium) or a negative control (5′-UUCUCCGAACGUGUCACGU-3′, Eurogentec, Liège, Belgium). Sponges were harvested for mRNA and proteins were analyzed after 28 or 42 days of culture. eUCB-MSCs monolayers cultured with expansion medium were used as a control (day zero, D0).

### 4.7. RNA Isolation and RT-qPCR

After culture, the total RNA of cells seeded in flasks for senescence analysis and of sponges seeded with cells were extracted using Trizol Reagent according to the manufacturer’s instructions. One microgram of total RNA was reverse transcribed into cDNA using reverse transcriptase (MMLV, Invitrogen Life Technologies, Carlsbad, CA, USA) and oligodT (Eurogentec, Liège, Belgium). Quantitative PCR was performed on a StepOne Plus Real-Time PCR System (Applied Biosystems, Foster City, CA, USA) using Power SYBR Green PCR (Applied Biosystems). Sequences of the primers and probe used are listed in Table 2. Relative gene expression was calculated using the 2^−ΔΔ*C*t^ method expressed as the mean of triplicate samples. Each sample was normalized to *β-Actin* and each group was normalized to the expression of undifferentiated eUCB-MSCs at passage one cultured without FGF-2 for the senescence analysis or to the expression levels of undifferentiated eUCB-MSCs at passage four for the induction of chondrogenesis in the 3D scaffold and to the expression levels of eUCB-MSCs cultured in sponges with ICM for 28 or 42 days for experiments of gene silencing.

### 4.8. Western Blots

After culture, seeded sponges and cells cultured in monolayers as controls were rinsed with PBS, crushed, and total proteins were extracted using the RIPA-lysis buffer with a protease inhibitor cocktail. The protein concentration was assessed according to the Bradford colorimetric procedure (Bio-Rad, Hercules, CA, USA). Then, 10 µg of total proteins were separated in 8% polyacrylamide gels containing 0.1% SDS and transferred to a polyvinylidene difluoride membrane (PVDF, Millipore, Billerica, MA, USA). Unspecific binding sites of the membrane were blocked with 10% non-fat milk powder in Tris-buffered saline with 0.1% Tween (TBST) for 1 h. Then, membranes were incubated overnight at 4 °C with rabbit anti-human type I collagen (Novotec, Bron, France), rabbit anti-human type II collagen (Novotec, Bron, France), rabbit anti-human type II B collagen (Covalab, Villeurbanne, France), rabbit anti-human type X collagen (Abcam, Cambridge, UK), rabbit anti-human HtrA1 (Merck Millipore, Billerica, MA, USA), or rabbit anti-human β-Tubulin (Santa Cruz Biotechnology, Dallas, TX, USA). Concerning the anti-human type IIB collagen, its generation was made by Covalab using exactly the same strategy as the one used by Aubert-Foucher et al. [61,62]. This antibody detects the pro α1(II)B and pN α1(II)B isoforms of type II collagen, according to the epitope targeted. The following day, membranes were washed three times, followed by incubation with HRP (HorseRadish Peroxidase)-conjugated goat anti-rabbit IgG antibody (Jackson Immunoresearch, West Grove, PA, USA). Signals were visualized with the chemiluminescence method (Western Lightning^®^ Plus-ECL, PerkinElmer, Waltham, Mass, USA) and developed on X-ray film (Santa Cruz Biotechnology, Dallas, TX, USA).

### 4.9. Statistical Analysis

All experiments were repeated at least three or four times with cells from different foals. Values are reported as box plots, floating bars with median or means ± SD. Statistical analyses were performed using the paired or multiple *t*-test for proliferation capacity and senescence analysis. Statistically significant differences between time points with the same culture medium were determined using the Friedman test. Statistically significant differences between the culture medium at the same time point were determined using the two-tailed Mann–Whitney test or Kruskall–Wallis test if more than two culture media were compared. Comparison of the differences between normoxia and hypoxia was performed using the two-tailed Mann–Whitney test. For gene silencing experiments, statistical analyses were performed using the Student’s *t*-test. All statistical analyses were conducted using GraphPad Prism 6 (GraphPad Software, Inc., San Diego, CA, USA). A *p*-value of ≤0.05 was considered to be significant.

## Figures and Tables

**Table 1:** Antibody panel for flow cytometric analysis.

Antibodies	Isotype	Clone	Flurochrome	Source
Anti-CD29	IgG1, κ	TS2/16	APC (Allophycocyanin)	BioLegend (San Diego, CA, USA)
Anti-CD44	IgG1, κ	J.173	PE (Phycoerythrin)	IOTest (Brea, CA, USA)
Anti-CD45	IgG2a	F10-89-4	Pacific Blue^®^	AbD Serotec (Kidlington, UK)
Anti-CD73	IgG1	10f1	APC	Abcam (Cambridge, UK)
Anti-CD90	IgM	DH24A	FITC (Fluorescein isothiocyanate)	InvestCare (London, UK)
Anti-CD105	IgG1	SN6	FITC	Abcam (Cambridge, UK)
Anti-type II MHC	IgG1	CVS20	RPE	AbD Serotec (Kidlington, UK)
Goat anti-mouse IgG1	IgG	Polyclonal	APC	Abcam (Cambridge, UK)
Goat anti-mouse IgM	IgG	Polyclonal	FITC	AbD Serotec (Kidlington, UK)

**Table 2:** Primers used for RT-qPCR.

Genes	Primer Sequence (5′-3′)
*β-Actin*	Foward: AGGCACCAGGGCGTGAT
	Reverse: CTCTTGCTCTGGGCCTCGT
*Col2a1*	Foward: GGCAATAGCAGGTTCACGTACA
	Reverse: CGATAACAGTCTTGCCCCACTT
*Col1a1*	Foward: TGCCGTGACCTCAAGATGTG
	Reverse: CGTCTCCATGTTGCAGAAGA
*Col9a1*	Foward: CCAAGAGGCCCAATCGACAT
	Reverse: GGGGAAGTCCGTTATCCTGG
*HtrA1*	Foward: GGACTTCATGTTTCCCTCAA
	Reverse: GTTCTGCTGAACAAGCAACA
*Acan*	Foward: TGTCAACAACAATGCCCAAGAC
	Reverse: CTTCTTCCGCCCAAAGGTCC
*Snorc*	Foward: TTTACCAGCTCAGTCCTCGG
	Reverse: CAGACAGAGAGCCATCCTGG
*Col10a1*	Foward: GCACCCCAGTAATGTACACCTATG
	Reverse: GAGCCACACCTGGTCATTTTC
*Osteocalcin (Ostc or BGLAP gene)*	Foward: AGAGTCTGGCAGAGGTGCAG
	Reverse: TCGTCACAGTCTGGGTTGAG
*Mmp3*	Foward: GAGGAAATGAGGAACAAGCGG
	Reverse : GAGGGAAACCCAGAGTGTGGA
*Mmp1*	Foward: CGAAGGGAACCCTCGGTGGGA
	Reverse: TGGCCTGGTCCACATCTGCTC
*Mmp13*	Foward: TGAAGACCCGAACCCTAAACAT
	Reverse: GAAGACTGGTGATGGCATCAAG
*Col11a1*	Foward: TTGCTGATGGGAAGTGGCAT
	Reverse: GCTGCTTTGGGGTCACCTAT
*Runx2*	Foward: GCAGTTCCCAAGCATTTCAT
	Reverse : CACTCTGGCTTTGGGAAGAG
*Col1a2*	Foward: CCAGAGTGGAGCAGCGGTTA
	Reverse: GGGATGTTTTCAGGTTGAGCC
*p53*	Foward: CACCTGAGGTTGGCTCTGAC
	Reverse: GCACAAACACGCACCTCAAA
*p21*	Foward: CTTGAAGTGGGCACAGCCTA
	Reverse: AAGTGCAGAGGAAGCCAACA
*Ki67*	Foward: AAGCTGCACGTTCATGGAGA
	Reverse: ACCCACAGTTCTTCCTCCGA
*Pcna*	Foward: GCGTGAACCTCACCAGTATGT
	Reverse: GCAAATTCGCCAGAAGGCAT
*Oct4*	Foward: AGTGAGAGGCAACCTGGAGA
	Reverse: ATACCGGTCCCCCTGAGAAA
*Fgf1*	Foward: TTGTACGGCTCACAGACACC
	Reverse: TTAGTCAGAGGAGACGGGCA
*Fgf2*	Foward: CAAACTACAACTTCAAGCAGAAGAGAGA
	Reverse: CCAGTAACCTTCCATCTTCCTTCAT

## Supplementary Materials

Supplementary materials can be found at http://www.mdpi.com/1422-0067/19/2/537/s1.

## References

[1] McIlwraith C.W., Frisbie D.D., Kawcak C.E. (2012). The horse as a model of naturally occurring osteoarthritis. Bone Jt. Res..

[2] Hardingham T.E. (2010). Fell-Muir lecture: Cartilage 2010—The known unknowns. Int. J. Exp. Pathol..

[3] Aigner T., Stöve J. (2003). Collagens—Major component of the physiological cartilage matrix, major target of cartilage degeneration, major tool in cartilage repair. Adv. Drug Deliv. Rev..

[4] Perkins N., Reid S., Morris R. (2005). Profiling the New Zealand Thoroughbred racing industry. 2. Conditions interfering with training and racing. N. Z. Vet. J..

[5] Aigner T., Cook J., Gerwin N., Glasson S., Laverty S., Little C., McIlwraith W., Kraus V. (2010). Histopathology atlas of animal model systems—Overview of guiding principles. Osteoarthr. Cartil..

[6] McIlwraith C.W., Fortier L.A., Frisbie D.D., Nixon A.J. (2011). Equine models of articular cartilage repair. Cartilage.

[7] Brittberg M., Lindahl A., Nilsson A., Ohlsson C., Isaksson O., Peterson L. (1994). Treatment of deep cartilage defects in the knee with autologous chondrocyte transplantation. N. Engl. J. Med..

[8] Nixon A.J., Begum L., Mohammed H.O., Huibregtse B., O’callaghan M.M., Matthews G.L. (2011). Autologous chondrocyte implantation drives early chondrogenesis and organized repair in extensive full- and partial-thickness cartilage defects in an equine model. J. Orthop. Res..

[9] Benya P.D., Padilla S.R., Nimni M.E. (1978). Independent regulation of collagen types by chondrocytes during the loss of differentiated function in culture. Cell.

[10] Friedenstein A.J., Gorskaja J.F., Kulagina N.N. (1976). Fibroblast precursors in normal and irradiated mouse hematopoietic organs. Exp. Hematol..

[11] Kobolak J., Dinnyes A., Memic A., Khademhosseini A., Mobasheri A. (2015). Mesenchymal stem cells: Identification, phenotypic characterization, biological properties and potential for regenerative medicine through biomaterial micro engineering of their niche. Methods.

[12] Zhou C., Yang B., Tian Y., Jiao H., Zheng W., Wang J., Guan F. (2011). Immunomodulatory effect of human umbilical cord Wharton’s jelly-derived mesenchymal stem cells on lymphocytes. Cell. Immunol..

[13] Murata D., Miyakoshi D., Hatazoe T., Miura N., Tokunaga S., Fujiki M., Nakayama K., Misumi K. (2014). Multipotency of equine mesenchymal stem cells derived from synovial fluid. Vet. J..

[14] Ortved K.F., Nixon A.J. (2016). Cell-based cartilage repair strategies in the horse. Vet. J..

[15] Dominici M., Le Blanc K., Mueller I., Slaper-Cortenbach I., Marini F., Krause D., Deans R., Keating A., Prockop D., Horwitz E. (2006). Minimal criteria for defining multipotent mesenchymal stromal cells. The International Society for Cellular Therapy position statement. Cytotherapy.

[16] Tessier L., Bienzle D., Williams L.B., Koch T.G. (2015). Phenotypic and Immunomodulatory Properties of Equine Cord Blood-Derived Mesenchymal Stromal Cells. PLoS ONE.

[17] De Schauwer C., Goossens K., Piepers S., Hoogewijs M.K., Govaere J.L.J., Smits K., Meyer E., Van Soom A., Van de Walle G.R. (2014). Characterization and profiling of immunomodulatory genes of equine mesenchymal stromal cells from non-invasive sources. Stem Cell Res. Ther..

[18] Stenderup K., Justesen J., Clausen C., Kassem M. (2003). Aging is associated with decreased maximal life span and accelerated senescence of bone marrow stromal cells. Bone.

[19] Kern S., Eichler H., Stoeve J., Klüter H., Bieback K. (2006). Comparative analysis of mesenchymal stem cells from bone marrow, umbilical cord blood, or adipose tissue. Stem Cells.

[20] Jin H.J., Bae Y.K., Kim M., Kwon S.-J., Jeon H.B., Choi S.J., Kim S.W., Yang Y.S., Oh W., Chang J.W. (2013). Comparative analysis of human mesenchymal stem cells from bone marrow, adipose tissue, and umbilical cord blood as sources of cell therapy. Int. J. Mol. Sci..

[21] Perrier E., Ronzière M.-C., Bareille R., Pinzano A., Mallein-Gerin F., Freyria A.-M. (2011). Analysis of collagen expression during chondrogenic induction of human bone marrow mesenchymal stem cells. Biotechnol. Lett..

[22] Handorf A.M., Li W.-J. (2011). Fibroblast growth factor-2 primes human mesenchymal stem cells for enhanced chondrogenesis. PLoS ONE.

[23] Chen X., Zhang F., He X., Xu Y., Yang Z., Chen L., Zhou S., Yang Y., Zhou Z., Sheng W. (2013). Chondrogenic differentiation of umbilical cord-derived mesenchymal stem cells in type I collagen-hydrogel for cartilage engineering. Injury.

[24] Mauck R.L., Yuan X., Tuan R.S. (2006). Chondrogenic differentiation and functional maturation of bovine mesenchymal stem cells in long-term agarose culture. Osteoarthr. Cartil..

[25] Freyria A.-M., Mallein-Gerin F. (2012). Chondrocytes or adult stem cells for cartilage repair: The indisputable role of growth factors. Injury.

[26] Kisiday J.D., Kopesky P.W., Evans C.H., Grodzinsky A.J., McIlwraith C.W., Frisbie D.D. (2008). Evaluation of adult equine bone marrow- and adipose-derived progenitor cell chondrogenesis in hydrogel cultures. J. Orthop. Res..

[27] Indrawattana N., Chen G., Tadokoro M., Shann L.H., Ohgushi H., Tateishi T., Tanaka J., Bunyaratvej A. (2004). Growth factor combination for chondrogenic induction from human mesenchymal stem cell. Biochem. Biophys. Res. Commun..

[28] Ronzière M.C., Perrier E., Mallein-Gerin F., Freyria A.-M. (2010). Chondrogenic potential of bone marrow- and adipose tissue-derived adult human mesenchymal stem cells. Biomed. Mater. Eng..

[29] Murphy M.K., Huey D.J., Hu J.C., Athanasiou K.A. (2014). TGF-β1, GDF-5, and BMP-2 stimulation induces chondrogenesis in expanded human articular chondrocytes and marrow-derived stromal cells. Stem Cells.

[30] Gomez-Leduc T., Hervieu M., Legendre F., Bouyoucef M., Gruchy N., Poulain L., de Vienne C., Herlicoviez M., Demoor M., Galera P. (2016). Chondrogenic commitment of human umbilical cord-blood derived mesenchymal stem cells in collagen matrices for cartilage engineering. Sci. Rep..

[31] Kafienah W., Mistry S., Dickinson S.C., Sims T.J., Learmonth I., Hollander A.P. (2007). Three-dimensional cartilage tissue engineering using adult stem cells from osteoarthritis patients. Arthritis Rheumatol..

[32] Gardner O.F.W., Archer C.W., Alini M., Stoddart M.J. (2013). Chondrogenesis of mesenchymal stem cells for cartilage tissue engineering. Histol. Histopathol..

[33] Cooke M.E., Allon A.A., Cheng T., Kuo A.C., Kim H.T., Vail T.P., Marcucio R.S., Schneider R.A., Lotz J.C., Alliston T. (2011). Structured three-dimensional co-culture of mesenchymal stem cells with chondrocytes promotes chondrogenic differentiation without hypertrophy. Osteoarthr. Cartil..

[34] Kang J.-G., Park S.-B., Seo M.-S., Kim H.-S., Chae J.-S., Kang K.-S. (2013). Characterization and clinical application of mesenchymal stem cells from equine umbilical cord blood. J. Vet. Sci..

[35] McIlwraith C.W., Frisbie D.D., Rodkey W.G., Kisiday J.D., Werpy N.M., Kawcak C.E., Steadman J.R. (2011). Evaluation of intra-articular mesenchymal stem cells to augment healing of microfractured chondral defects. Arthrosc. J. Arthrosc. Relat. Surg..

[36] Pigott J.H., Ishihara A., Wellman M.L., Russell D.S., Bertone A.L. (2013). Investigation of the immune response to autologous, allogeneic, and xenogeneic mesenchymal stem cells after intra-articular injection in horses. Vet. Immunol. Immunopathol..

[37] Williams L.B., Koenig J.B., Black B., Gibson T.W.G., Sharif S., Koch T.G. (2016). Equine allogeneic umbilical cord blood derived mesenchymal stromal cells reduce synovial fluid nucleated cell count and induce mild self-limiting inflammation when evaluated in an LPS induced synovitis model. Equine Vet. J..

[38] Wilke M.M., Nydam D.V., Nixon A.J. (2007). Enhanced early chondrogenesis in articular defects following arthroscopic mesenchymal stem cell implantation in an equine model. J. Orthop. Res..

[39] Hillmann A., Ahrberg A.B., Brehm W., Heller S., Josten C., Paebst F., Burk J. (2016). Comparative characterization of human and equine mesenchymal stromal cells: A basis for translational studies in the equine model. Cell Transplant..

[40] Koch T.G., Heerkens T., Thomsen P.D., Betts D.H. (2007). Isolation of mesenchymal stem cells from equine umbilical cord blood. BMC Biotechnol..

[41] Koch T.G., Thomsen P.D., Betts D.H. (2009). Improved isolation protocol for equine cord blood-derived mesenchymal stromal cells. Cytotherapy.

[42] Bieback K., Kern S., Klüter H., Eichler H. (2004). Critical parameters for the isolation of mesenchymal stem cells from umbilical cord blood. Stem Cells.

[43] Zhang X., Hirai M., Cantero S., Ciubotariu R., Dobrila L., Hirsh A., Igura K., Satoh H., Yokomi I., Nishimura T. (2011). Isolation and characterization of mesenchymal stem cells from human umbilical cord blood: Reevaluation of critical factors for successful isolation and high ability to proliferate and differentiate to chondrocytes as compared to mesenchymal stem cells from bone marrow and adipose tissue. J. Cell. Biochem..

[44] Solchaga L.A., Penick K., Goldberg V.M., Caplan A.I., Welter J.F. (2010). Fibroblast growth factor-2 enhances proliferation and delays loss of chondrogenic potential in human adult bone-marrow-derived mesenchymal stem cells. Tissue Eng. Part A.

[45] Wagner W., Horn P., Castoldi M., Diehlmann A., Bork S., Saffrich R., Benes V., Blake J., Pfister S., Eckstein V. (2008). Replicative senescence of mesenchymal stem cells: A continuous and organized process. PLoS ONE.

[46] Pittenger M.F., Mackay A.M., Beck S.C., Jaiswal R.K., Douglas R., Mosca J.D., Moorman M.A., Simonetti D.W., Craig S., Marshak D.R. (1999). Multilineage potential of adult human mesenchymal stem cells. Science.

[47] Barberini D.J., Freitas N.P.P., Magnoni M.S., Maia L., Listoni A.J., Heckler M.C., Sudano M.J., Golim M.A., da Cruz Landim-Alvarenga F., Amorim R.M. (2014). Equine mesenchymal stem cells from bone marrow, adipose tissue and umbilical cord: Immunophenotypic characterization and differentiation potential. Stem Cell Res. Ther..

[48] De Schauwer C., Piepers S., Van de Walle G.R., Demeyere K., Hoogewijs M.K., Govaere J.L.J., Braeckmans K., Van Soom A., Meyer E. (2012). In search for cross-reactivity to immunophenotype equine mesenchymal stromal cells by multicolor flow cytometry. Cytom. Part A.

[49] Vieira N.M., Brandalise V., Zucconi E., Secco M., Strauss B.E., Zatz M. (2010). Isolation, characterization, and differentiation potential of canine adipose-derived stem cells. Cell Transplant..

[50] Braun J., Hack A., Weis-Klemm M., Conrad S., Treml S., Kohler K., Walliser U., Skutella T., Aicher W.K. (2010). Evaluation of the osteogenic and chondrogenic differentiation capacities of equine adipose tissue-derived mesenchymal stem cells. Am. J. Vet. Res..

[51] Hackett C.H., Flaminio M.J.B.F., Fortier L.A. (2011). Analysis of CD14 Expression levels in putative mesenchymal progenitor cells isolated from equine bone marrow. Stem Cells Dev..

[52] Iacono E., Merlo B., Romagnoli N., Rossi B., Ricci F., Spadari A. (2015). Equine bone marrow and adipose tissue mesenchymal stem cells: cytofluorimetric characterization, in vitro differentiation, and clinical application. J. Equine Vet. Sci..

[53] Kopesky P.W., Lee H.Y., Vanderploeg E.J., Kisiday J.D., Frisbie D.D., Plaas A.H.K., Ortiz C., Grodzinsky A.J. (2010). Adult equine bone-marrow stromal cells produce a cartilage-like ECM mechanically superior to animal-matched adult chondrocytes. Matrix Biol..

[54] Co C., Vickaryous M.K., Koch T.G. (2014). Membrane culture and reduced oxygen tension enhances cartilage matrix formation from equine cord blood mesenchymal stromal cells in vitro. Osteoarthr. Cartil..

[55] Ranera B., Remacha A.R., Álvarez-Arguedas S., Castiella T., Vázquez F.J., Romero A., Zaragoza P., Martín-Burriel I., Rodellar C. (2013). Expansion under hypoxic conditions enhances the chondrogenic potential of equine bone marrow-derived mesenchymal stem cells. Vet. J..

[56] Kisiday J.D., Goodrich L.R., McIlwraith C.W., Frisbie D.D. (2013). Effects of equine bone marrow aspirate volume on isolation, proliferation, and differentiation potential of mesenchymal stem cells. Am. J. Vet. Res..

[57] Duval E., Leclercq S., Elissalde J.-M., Demoor M., Galera P., Boumediene K. (2009). Hypoxia-inducible factor 1α inhibits the fibroblast-like markers type I and type III collagen during hypoxia-induced chondrocyte redifferentiation. Arthritis Rheumatol..

[58] Galera P., Rédini F., Vivien D., Bonaventure J., Penfornis H., Loyau G., Pujol J.-P. (1992). Effect of transforming growth factor-beta 1 (TGF-beta 1) on matrix synthesis by monolayer cultures of rabbit articular chondrocytes during the dedifferentiation process. Exp. Cell Res..

[59] Yang B., Guo H., Zhang Y., Chen L., Ying D., Dong S. (2011). MicroRNA-145 regulates chondrogenic differentiation of mesenchymal stem cells by targeting SOX9. PLoS ONE.

[60] Legendre F., Ollitrault D., Hervieu M., Baugé C., Maneix L., Goux D., Chajra H., Mallein-Gerin F., Boumediene K., Galera P. (2013). Enhanced hyaline cartilage matrix synthesis in collagen sponge scaffolds by using siRNA to stabilize chondrocytes phenotype cultured with bone morphogenetic protein-2 under hypoxia. Tissue Eng. Part C Methods.

[61] Aubert-Foucher E., Mayer N., Pasdeloup M., Pagnon A., Hartmann D., Mallein-Gerin F. (2014). A unique tool to selectively detect the chondrogenic IIB form of human type II procollagen protein. Matrix Biol..

[62] Branly T., Contentin R., Desancé M., Jacquel T., Bertoni L., Jacquet T., Mallein-Gerin F., Denoix J.-M., Audigié F., Demoor M. (2018). Improvement of the chondrocyte-specific phenotype upon equine bone marrow mesenchymal stem cell differentiation. Influence of culture time, transforming growth factors and type I collagen siRNAs on the differentiation index. Int. J. Mol. Sci..

