# Supplementary material for: Chondrogenic Differentiation of Defined Equine Mesenchymal Stem Cells Derived from Umbilical Cord Blood for Use in Cartilage Repair Therapy
Source: Int J Mol Sci. 2018 Feb 10;19(2):537. doi: 10.3390/ijms19020537 (PMC5855759; doi:10.3390/ijms19020537)
Supplement: Supplementary file 1 [file ijms-19-00537-s001.pdf]

Chondrogenic differentiation of defined equine mesenchymal stem cells derived from umbilical cord blood for use in cartilage repair therapy.

Mélanie Desancé[1], Romain Contentin[1], Lélia Bertoni[2], Tangni Gomez-Leduc[1], Thomas Branly[1], Sandrine Jacquet[2], Jean-Marc Betsch[3], Agnès Batho[1,4], Florence Legendre[1], Fabrice Audigié[2], Philippe Galéra[1*†] & Magali Demoor[1*].

* Contributed equally

† Corresponding author: philippe.galera@unicaen.fr, magali.demoor@unicaen.fr

1. NORMANDIE UNIV, UNICAEN, BIOTARGEN, 14000 CAEN, FRANCE.

2. Center of Imaging and Research on Locomotor Affections in Equines, Ecole Vétérinaire d'Alfort, Université Paris-Est, 14430 Goustranville, France.

3. Clinique Vétérinaire Equine de Méheudin, Méheudin, 61150 Ecouché, France.

4. EFS Caen, 14000 Caen, France.

	P2	P3	P4	P5
Number of different samples	20	16	7	4
Total of cells (millions)	239.6	234.88	93.64	43.7

Table S1. Cryopreservation of mesenchymal stem cells. Equine umbilical cord blood-derived mesenchymal stem cells banking at different passages.

A

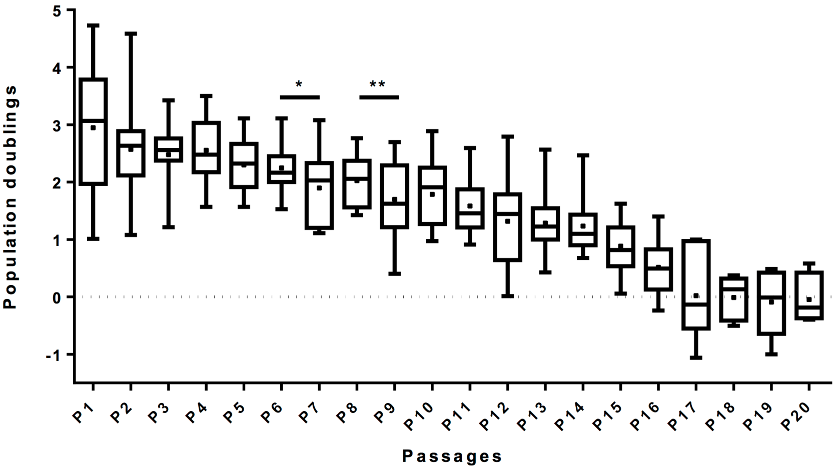

B

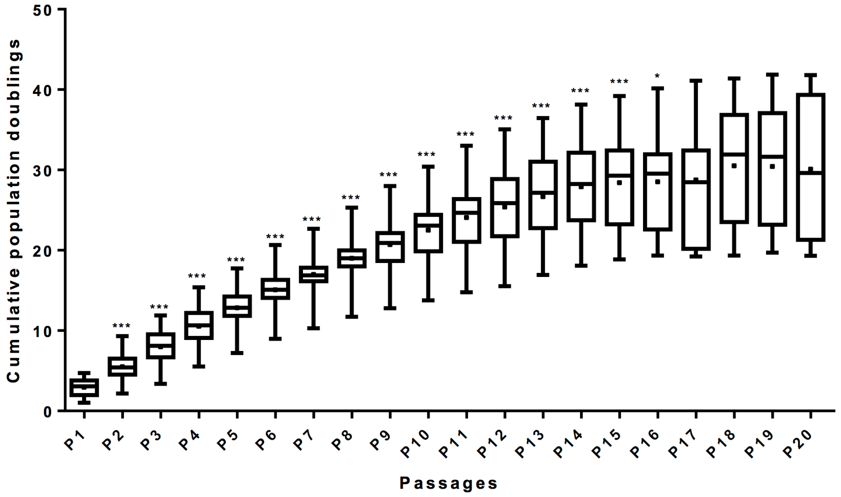

Figure S1. Analysis of proliferation capacity of equine umbilical cord blood-derived mesenchymal stem cells (eUCB-MSCs). (**A**) Mean value of population doublings and (**B**) mean value of cumulative population doublings. Population doublings were determined at each passage of the adherent eUCB-MSCs cultured in the absence of FGF-2. Graph represents mean ± standard deviation (n=12 between P1 and P14, n=10 for P15, n=9 for P16, n=7 for P17, n=5 for P18 and P19, n=4 for P20). Statistically significant differences among eUCB-MSCs for two successive passages were determined using paired t test (*p<0.05, **p<0.01, ***p<0.001).
